# Biophysical Studies and In Vitro Effects of Tumor Cell Lines of Cannabidiol and Its Cyclodextrin Inclusion Complexes

**DOI:** 10.3390/pharmaceutics14040706

**Published:** 2022-03-26

**Authors:** Kyriaki Hatziagapiou, Kostas Bethanis, Eleni Koniari, Elias Christoforides, Olti Nikola, Athena Andreou, Aimilia Mantzou, George P. Chrousos, Christina Kanaka-Gantenbein, George I. Lambrou

**Affiliations:** 1 Choremeio Research Laboratory, First Department of Pediatrics, National and Kapodistrian University of Athens, Thivon & Levadeias 8, 11527 Athens, Greece; khatziag@med.uoa.gr (K.H.); onikola@med.uoa.gr (O.N.); amantzou@med.uoa.gr (A.M.); ckanaka@med.uoa.gr (C.K.-G.); 2 Division of Endocrinology, First Department of Pediatrics, Metabolism, and Diabetes, National and Kapodistrian University of Athens, Thivon & Levadeias 8, 11527 Athens, Greece; 3 Physiotherapy Department, Faculty of Health and Care Sciences, State University of West Attica, Agiou Spiridonos 28, 12243 Athens, Greece; 4 Physics Laboratory, Department of Biotechnology, School of Applied Biology and Biotechnology, Agricultural University of Athens, 75 Iera Odos, 11855 Athens, Greece; el_chr@aua.gr; 5 UNESCO Chair on Adolescent Health Care, “Aghia Sophia” Children’s Hospital, University Research Institute of Maternal and Child Health & Precision Medicine, National and Kapodistrian University of Athens, Thivon & Levadeias 8, 11527 Athens, Greece; hkoniari@med.uoa.gr (E.K.); chrousge@med.uoa.gr (G.P.C.); 6 Genetics Laboratory, Department of Biotechnology, School of Applied Biology and Biotechnology, Agricultural University of Athens, 75 Iera Odos, 11855 Athens, Greece; aandre@aua.gr

**Keywords:** cannabidiol (CBD), glioblastoma, rhabdomyosarcoma, cyclodextrin, inclusion complexes, X-ray crystallography, phase solubility, molecular dynamics

## Abstract

Phytocannabinoids possess anticancer properties, as established in vitro and in vivo. However, they are characterized by high lipophilicity. To improve the properties of cannabidiol (CBD), such as solubility, stability, and bioavailability, CBD inclusion complexes with cyclodextrins (CDs) might be employed, offering targeted, faster, and prolonged CBD release. The aim of the present study is to investigate the in vitro effects of CBD and its inclusion complexes in randomly methylated *β*-CD (RM-*β*-CD) and 2-hyroxypropyl-*β*-CD (HP-*β*-CD). The enhanced solubility of CBD upon complexation with CDs was examined by phase solubility study, and the structure of the inclusion complexes of CBD in 2,6-di-O-methyl-*β*-CD (DM-*β*-CD) and 2,3,6-tri-O-methyl-*β*-CD (TM-*β*-CD) was determined by X-ray crystallography. The structural investigation was complemented by molecular dynamics simulations. The cytotoxicity of CBD and its complexes with RM-*β*-CD and HP-*β*-CD was tested on two cell lines, the A172 glioblastoma and TE671 rhabdomyosarcoma cell lines. Methylated *β*-CDs exhibited the best inclusion ability for CBD. A dose-dependent effect of CBD on both cancer cell lines and improved efficacy of the CBD–CDs complexes were verified. Thus, cannabinoids may be considered in future clinical trials beyond their palliative use as possible inhibitors of cancer growth.

## 1. Introduction

Cannabinoids, such as dronabinol and nabilone, have long been used in association with chemotherapy in order to relieve its side effects, i.e., weight loss, nausea, and vomiting, and mitigate cancer pain; however, their use is still limited due to their psychoactive side effects, as they contain the active component of marijuana, delta(Δ)9-tetrahydrocannabinol (THC). However, new studies suggest that their activity can be ascribed not solely to these “palliative” effects but rather they could possess some interesting anti-cancer properties, e.g., antioxidant properties, attenuation of tumor cell proliferation, sensitization of cancer cells to apoptosis, inhibition of tumor cell migration and invasion, and inversion of chemotherapy drug resistance [1,2,3,4,5].

**Figure 1:**
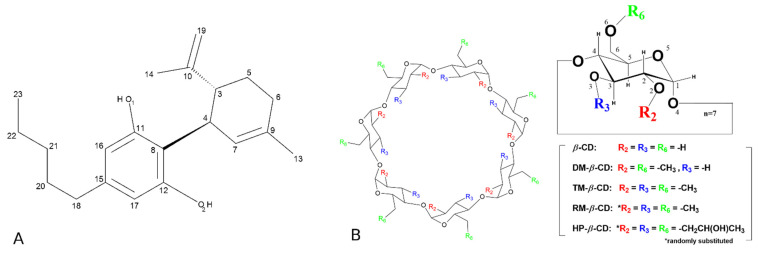
(**A**) Schematic representation of the chemical structure of cannabidiol (CBD) and (**B**) schematic representation of native *β*-cyclodextrin (*β*-CD), heptakis(2,6-di-*O*-methyl)-*β*-cyclodextrin (DM-*β*-CD), heptakis(2,3,6-tri-*O*-methyl)-*β*-cyclodextrin (TM-*β*-CD), randomly-methylated-*β*-cyclodextrin (RM-*β*-CD), and 2-hydroxypropyl-*β*-cyclodextrin (HP-*β*-CD).

Compounds extracted from plants still provide some of the most original and promising approaches for discovering new drugs [6]. Under this perspective, cannabidiol (CBD; C_21_ H_30_ O_2_) (Figure 1A) has been found to be endowed with anticancer, antioxidant, and genome-protective properties as established in vitro in several malignant cell lines derived mainly from adult patients and in vivo experiments [7,8,9,10]. Furthermore, the current literature does not provide proof that cannabinoids decrease efficacies of clinically used chemotherapeutics, but studies rather suggest that cannabinoids enhance their anti-tumorigenic properties, while counteracting some of the adverse effects of chemotherapy [11,12,13]. Despite this, the bioavailability of oral CBD is considered low due to poor aqueous solubility, erratic gastrointestinal absorption and significant first-pass metabolism, hampering its therapeutic potential and resulting in a variable pharmacokinetic profile [14,15]. In this context, CBD can take great advantage of nanomedicine-based formulation strategies. To improve the solubility and bioavailability of poorly water-soluble drugs, several methods have been developed, such as solid dispersion, complexation, lipid-based systems, micronization, nanonization, and co-crystals [16]. Among these, solid dispersion by inclusion complex formation in biodegradable natural polymers is one of the most potent and successful methods. Cyclodextrins (CDs), frequently micronized with different techniques [17,18], are widely used as molecular carriers (hosts) for complexation of drugs, as it is generally accepted that the inclusion of bioactive compounds in CDs could improve their bioavailability, enhance their stability, and considerably reduce the side effects following administration [19,20].

Cyclodextrins are naturally occurring cyclic oligosaccharides, formed by enzymatic degradation of starch. The three main types of native CDs, *α*-, *β*-, and *γ*-CDs consist of 6, 7, and 8 α-1,4-linked d-glucopyranose units, respectively. Due to their truncated cone shape, with a hydrophilic exterior and a hydrophobic cavity, CDs have the ability to encapsulate hydrophobic molecules or parts of molecules inside their cavity, forming non-covalent dynamic inclusion complexes in aqueous solution. As a result of this inclusion, a significant improvement of the physicochemical properties and bioavailability of the guest drugs is usually observed, and in addition to the excellent biocompatibility of the CDs, these inclusion complexes have gained an important role in the pharmaceutical industry [21].

The present research endeavors to study the effects of CBD and its inclusion complexes with randomly methylated *β*-CD (RM-*β*-CD) and 2-hyroxypropyl-*β*-CD (HP-*β*-CD) (Figure 1B) derivatives on malignant cell lines in terms of cytotoxicity. In addition, the physicochemical and structural characteristics of CBD inclusion complexes in these CDs are investigated using physicochemical methods, such as phase solubility study and X-ray crystallography complemented by theoretical (molecular dynamics simulations) studies. The safety of these CDs on neuroblastoma cell lines and possible antitumor action of HP-*β*-CD per se has been demonstrated in a previous work [22].

The inclusion of CBD in native *α*-, *β*-, and *γ*-CDs has been studied by various spectroscopic and calorimetric techniques including phase solubility studies, dissolution studies, Gas-Chromatography–Mass Spectroscopy (GCMS), Thermal Gravimetric Analysis (TGA), Powder X-Ray Diffraction (PXRD), and Scanning Electron Microscopy (SEM) [23,24,25]. Moreover, the inclusion of CBD in CD derivatives such as 2,6-di-O-methyl-*β*-CD (DM-*β*-CD), randomly methylated *β*-CD (RM-*β*-CD), 2-hydroxypropyl-*β*-, and *γ*-CD (HP-*β*-CD and HP-*γ*-CD) has been investigated by phase solubility studies, Differential Scanning Calorimetry (DSC), and ^1^ H-NMR [26,27]. All these methods have manifested strong evidence of the formation of inclusion complexes, indicating host–guest ratios, illustrating intermolecular interactions, estimating complex affinities, and providing structural insight into the inclusion mode. However, no direct and detailed structural information, such as that provided by the determination of the crystal structure of the inclusion complexes, has been depicted so far.

In the present study, the structure of the inclusion compounds of CBD with 2,6-di-O-methyl-*β*-CD (DM-*β*-CD) and 2,3,6-tri-O-methyl-*β*-CD (TM-*β*-CD) (Figure 1B) was determined by X-ray crystallography and thoroughly examined to reveal host–guest stoichiometry, inclusion geometry and intermolecular interactions in the crystalline state. The structural details of guest inclusion in CDs are valuable in the engineering of modified guest–host preparations with optimized pharmacological properties. However, crystallographic structures of guest–CD complexes provide structures that are affected by crystal contacts and constitute averages of subsets of possible guest-binding modes. Thus, complementary studies of the dynamical behavior and the stability of these complexes in aqueous environment were conducted based on molecular dynamics (MD) simulations, which is a commonly used method to study CD complexes [28]. MD studies were based on the crystallographically determined or docked models, and consequent calculations of the binding affinities of these inclusion complexes by the Molecular Mechanics/Generalized Born Surface Area (MM/GBSA) method were used to evaluate the stability and inspect the dynamic behavior of the inclusion complexes. Finally, phase solubility studies of CBD/*β*-CD, CBD/RM-*β*-CD, and CBD/HP-*β*-CD were conducted in order to estimate the inclusion complex type, the stability constant *K_C_*, and the Complexation Efficiency (CE) of these inclusion complexes, guiding the preparation of the solutions used in the cell cytotoxicity experiments.

The findings of the present work provide detailed information on CBD inclusion complexation with CDs and indicate possibly improved efficacy of the encapsulated drug in the cytotoxicity assays on the studied cancer cell lines.

## 2. Materials and Methods

### 2.1. Preparation, Characterization, and Structure Determination of the Inclusion Complexes

#### 2.1.1. Reagents

CBD as white crystals (Enecta Cc500 CBD crystals, 500 mg, 99% pure) were kindly offered by Hempoil^®^ (Athens, Greece) and Enecta Bv (Amsterdam, Netherlands). *β*-CD, DM-*β*-CD, TM-*β*-CD, RM-*β*-CD (degree of substitution (DS) ~ 12), and HP-*β*-CD (DS ~ 4.5) were purchased from CycloLab Ltd. (Budapest, Hungary).

#### 2.1.2. Preparation of CBD Standard Solutions and Construction of Calibration Curve

The UV–visible (UV–Vis) spectrophotometer (BioBase Group; BK-S380; Jinan, Shandong, China) was employed to evaluate the solubility of CBD and its inclusion complexes. The visible absorption maximum of the CBD was at 273 nm. Seven standards of CBD in methanol were measured, each three times, to obtain a calibration curve, and corresponding concentrations of 0.20, 0.40, 0.60, 0.80, 1.00, 1.20, and 1.50 mM were prepared. The calibration curve was depicted by plotting the absorbance against the above CBD concentrations and by applying the linear regression analysis according to a previously described procedure [29].

#### 2.1.3. Phase Solubility Measurements

Solubility studies were carried out according to the method reported by Higuchi & Connors (1965) [30]. Excess amount of CBD (50 mg) was added to 10 mL of deionized water containing various concentrations between 1 to 15 mM for *β*-CD and 1 to 60 mM for both RM-*β*-CD and HP-*β*–CD. The solutions were further mixed using an orbital shaker (PHOENIX Instrument Laboratory Shaker RS-OS 5; Berlin, Germany) at 25°C for 48 h to ensure equilibrium, and then the collected samples were filtered (0.45 μm), appropriately diluted with a 1:1 (*v/v*) methanol to water solution, and assayed using the BK-S380 UV–Vis spectrophotometer at 273 nm.

#### 2.1.4. Drug Preparation and Storage for In Vitro Tests

The CBD solution was prepared by dissolving the CBD crystals in DMSO aqueous solution (5% *v/v*) at a concentration of 4 mg/mL (12.7 mM).

The preparation of the CBD/CD solutions was guided by the results of the preceding phase solubility studies. According to the phase solubility diagrams and the complexation-efficiency (CE) estimations (see Section 3.1), a 4 mg/mL (12.7 mM) CBD concentration in aqueous solution can be achieved only in the presence of RM-*β*-CD or HP-*β*-CD. Thus, CBD/RM-*β*-CD and CBD/HP-*β*-CD solutions were prepared by mixing crystalline CBD in aqueous (dH_2_ O) CD solutions to final molar ratios of about 1:5 and 1:37, respectively. The mixtures were stirred at room temperature for 24 h and subsequently passed through a 0.45-μm filter. These stock solutions were mixed with cell culture media to a final CBD concentration of 0.570 mg/mL (1.8 mM), which was used for further serial dilutions up to 0.004 mg/mL (0.014 mM) and stored at −80 °C. The same procedure was followed to prepare CD control solutions [22].

#### 2.1.5. Crystallization

CBD in crystalline form was added to aqueous solutions of DM-*β*-CD or TM-*β*-CD (3 mM) at 1:1 host to guest mole ratios. The two mixtures were stirred for 1 h at room temperature and subsequently maintained at 321 K for a period of one week according to a previously described procedure [31]. Clear, light, colorless, rod-like, and prism-like crystals, suitable for X-ray data collection, were obtained in the case of CBD/DM-*β*-CD and CBD/TM-*β*-CD, respectively. The specimens were kept in paraffin oil until they were harvested for data collection.

#### 2.1.6. Single-Crystal X-ray Diffraction Experiments

X-ray diffraction data were collected at 100(2) K on a Bruker D8-VENTURE diffractometer, using CuKa radiation (λ = 1.54178 Å) and an Oxford Cryosystems (Oxford Cryosystems Ltd.; Long Handorough, UK) low-temperature device.

In the case of the CBD/DM-*β*-CD single crystal, a total of 3067 frames were collected with a total exposure time of 21.30 h. The frames were integrated with the Bruker SAINT Software package using a narrow-frame algorithm [32]. The integration of the data using an orthorhombic unit cell yielded a total of 199,206 reflections to a maximum *θ* angle of 50.95° (0.99 Å resolution). The final cell constants of *a* = 16.596(2) Å, *b* = 15.5514(18) Å, *c* = 33.269(4) Å, *β* = 90.026(8), and volume = 8586.6(18) Å^3^ were based upon the refinement of the XYZ-centroids of 9450 reflections above 20 σ(I) with 5.312° < 2*θ* < 100.7°. Data were corrected for absorption effects using the Multi-Scan method (SADABS) [33]. The ratio of minimum to maximum apparent transmission was 0.824.

In the case of the CBD/TM-*β*-CD single crystal, a total of 3423 frames were collected exposing the specimen for 21.64 h. The frames were integrated with the Bruker SAINT Software package using a narrow-frame algorithm. The integration of the data using a monoclinic unit cell yielded a total of 151,333 reflections to a maximum *θ* angle of 60.2° (0.89 Å resolution). The final cell constants of *a* = 15.360(2) Å, *b* = 27.955(4) Å, *c* = 21.201(3) Å, *β* = 110.963(7)°, and volume = 8501.(2) Å^3^ are based upon the refinement of the XYZ-centroids of 9683 reflections above 20 σ(I) with 5.469° < 2*θ* < 128.3°. Data were corrected for absorption effects using the Multi-Scan method (SADABS). The ratio of minimum to maximum apparent transmission was 0.816. Both structures were solved by Patterson-seeded dual-space recycling using the SHELXD program [34] and refined by full-matrix least squares against F^2^ using SHELXL-2014/7 [35] through the SHELXLE GUI [36], giving final R_1_ indexes of 0.1 and 0.06, respectively. Due to the structural complexity of the models, soft restraints on lengths and angles of the guest molecules were applied using the PRODRG2 webserver [37]. Anisotropic displacement parameters were refined using the soft restraint (SIMU) implemented in the SHELXL program where necessary. All H atoms of both host and guest molecules were placed geometrically for temperature of 100 K and allowed to ride on the parent atoms. U_iso_(H) values were assigned in the range 1.2–1.5 times U_eq_ of the parent atom. In an effort to maintain a high (>7.0) data to parameters ratio, anisotropic thermal parameters were imposed to selected, non-H atoms of the host molecules. In the CBD/DM-*β*-CD case, the structure was refined as an inversion twin, and extinction corrections have been also applied to the final solution. Twelve reflections that illustrated high divergence between the Fo and Fc values were omitted at the last stages of refinement in the case of the CBD/TM-*β*-CD structure.

Mercury, Pymol, and Olex2 were used to illustrate and geometrically analyze the crystal structures. Details of the crystallographic experiment and refinement are summarized in Table 1 [38,39,40]. Crystallographic information files with embedded structure factors have been deposited into the Cambridge Structural Database (CSD) under the deposition numbers CCDC: 2098419 and 2094890.

#### 2.1.7. Computational Studies

Τhe inclusion complexes of CBD in DM-*β*-CD, TM-*β*-CD, and HP-*β*-CD were studied by MD simulations. In the cases of the CBD/DM-*β*-CD and CBD/TM-*β*-CD complexes, the crystallographically determined structures were used as the starting 3 D models, whereas in the CBD/ HP-*β*-CD case, the starting models were generated by molecular docking using AutoDock Vina [41]. The subsequent MD simulations for the three aforementioned systems were performed using the AMBER 12 program [42] resulting in three single trajectories. The methylated and hydroxy-propylated glucopyranose units of the hosts were built and prepared with the programs VEGAZZ [43] and GAMESS [44]. Ante_RED-1.5.pl and RED-vIII.52.pl scripts [45] were run in order to obtain suitable files for the program PARMCHK implemented in AMBER 12. Modified CDs were treated with q4 md-CD force fields [46], respectively, while GAFF parameters and AM1 BCC charges were applied to the guest molecule using ANTECHAMBER. Each inclusion complex was initially immersed in a truncated octahedral box of TIP3 P waters forming a 10 Å thick water shell around the structure using xLEaP. Hydrogen atoms were also added with xLEaP in all systems.

MD calculations and minimizations were carried out with SANDER. Periodic boundary conditions were imposed by means of the particle mesh Ewald method using a 10-Å limit for the direct-space sum. The protocol included energy minimization for hydrogen atoms with positional restraints of 50 kcal mol^−1^ Å^−2^ on the non-hydrogen atoms, heating equilibration of the solvent in the canonical (NVT) ensemble using positional restraints and the Berendsen thermostat algorithm with coupling constants of 0.5 ps to control temperature and pressure, unrestrained energy minimization, gradual temperature increase from 5 to 300 K with 10 kcal mol^−1^ Å^−2^ restraints on the atoms of the inclusion complex, gradual release of the restraints in successive steps at 300 K, and density equilibration in the isobaric–isothermal (NPT) ensemble for 250 ps. Subsequently, production runs using a Berendsen-type algorithm with coupling constants of 1.0 ps were carried out under physiological conditions for additional 20 ns in the NPT ensemble. Root-mean-square deviation (RMSD) calculations as well as geometric analyses of the MD trajectories were performed by CPPTRAJ [47]. Visualization of structures was carried out with UCSF Chimera [48].

The binding affinities (in kcal/mol) and their standard deviations for the examined inclusion complexes were estimated by using the MM/GBSA method [49]. MM/GBSA is a post-processing approach which computes the Gibbs free energies of molecules in solution from the produced MD trajectories. The method combines the molecular mechanical (MM) energies with the continuum solvent approaches (Generalized Born model and surface area continuum solvation method (GBSA)) at a reasonable computational cost. The entropic penalty (Δ*S*) incurred upon guest binding was calculated by extracting snapshots from the MD trajectories every 100 frames using the NMODE module of AMBER and added to the enthalpic term according to:

(1) $$\Delta G_{Bind}=\Delta G_{\left(GB\right)}-T\cdot \Delta S$$

### 2.2. Biological Experiments

#### 2.2.1. Cell Culture and Reagents

A172 glioblastoma (ECACC 88062428) and TE-671 (ECACC 89071904) rhabdomyosarcoma cell lines were purchased from the European Collection of Cell Cultures (ECACC; London, UK). A172 cells were cultured in DMEM (Dulbecco’s Modified Eagle’s Medium) (Invitrogen; Carlsbad, CA, USA), supplemented with 1 g/L D-glucose and 110 mg/L sodium pyruvate, 1% penicillin/streptomycin (Invitrogen; Carlsbad, CA, USA), and 15% foetal bovine serum (FBS) (Invitrogen; Carlsbad, CA, USA). The TE-671 cell line was initially reported to originate from a cerebellar medulloblastoma before irradiation therapy of a six-year-old Caucasian female patient [50,51]. However, based on phenotypic characteristics, cytogenetic analysis, and the presence of an activated N-ras gene due to a relatively uncommon activating point mutation (p.Gln61 His), the TE671 cell line is considered parental or even identical to the RD rhabdomyosarcoma cell line, although several reports still refer to this cell line as medulloblastoma [52,53,54,55,56,57,58]. TE-671 cells were grown in DMEM supplemented with 2 mM L-glutamine, 10% FBS, and 1% penicillin/streptomycin. Cells were allowed to grow at ~80% confluence and harvested using 0.1% trypsin (Gibco™, Thermo Fisher Scientific; Oslo, Norway) and with centrifugation at 2400 rpm for 10′. Supernatant was discarded and pellet was kept for further processing. Cell population counts were determined with the use of a NIHON KOHDEN CellTaq-α hematology analyzer (Nihon Kohden EUROPE GmbH; Rosbach, Germany).

#### 2.2.2. Experimental Setup

Experiments were performed in 96-well plates (CellStar^®^; Sigma-Aldrich Chemie GmbH; Taufkirchen, Germany). Experimental setup included a column of cell culture medium only, a column of cell culture medium and the respective staining chemical, a column of cultured cells only (no drug, nor staining agent), and a column of cultured cells and the respective staining chemical, whereas the remaining wells were used for the testing of several concentrations of the testing agents. Wells containing cell culture medium only with staining agent were used as blank, whereas wells with cultured cells without any chemical compound (untreated cells) but with staining agent were used as positive controls.

#### 2.2.3. Assessment of Cell Proliferation and Viability

Assessment of cell viability after incubation with testing agents was performed with resazurin reduction experiments, using Alamar Blue viability assay (GIBCO^®^ Invitrogen Inc.; Carlsbad, CA, USA), as previously described [59,60]. In brief, cells were cultured in 96-well plates at a density of 2 × 10^4^ cells (*C* = 10^5^ cells/lt) per well in their respective medium. Cells were incubated for 24 h at 37 °C, until wells were confluent. Following 24 h, testing agents were added separately to the wells by serial dilution. More specifically, cells were exposed at 0 h to successive diluted concentrations of CBD in DMSO 5% to final concentrations of 0.57, 0.285, 0.143, 0.071, 0.036, 0.018, 0.009, and 0.0045 mg/mL and incubated for 24, 48, and 72 h. Additionally, cells were exposed to the same consecutive concentrations of CBD inclusion complexes with CDs: CBD/RM-*β*-CD and CBD/HP-*β*-CD and isolated CDs, RM-*β*-CD, and HP-*β*-CD.

To quantify the number of viable cells at each time point (24, 48, 72 h), Alamar Blue was added to wells, to a final concentration of 10%, and cells were incubated for 6 h. Thereafter, the reduced form of Alamar Blue was determined by fluorescence measurements at excitation wavelength 550 nm and emission of 590 nm. Measurements were carried out using the automated microtiter plate reader Victor^3^ (Perkin Elmer Inc.; Waltham, MA, USA). Signal intensities were normalized by subtracting the blank signal intensity from the signal intensity of the experimental wells. Each drug testing was carried out in triplicates and performed at least three independent times.

#### 2.2.4. Microscopy

Cells were microscopically observed on a fluorescent microscope (Zeiss AX10; Carl Zeiss AG; Jena, Germany) equipped with a AxioCam ICm1 ZEISS camera at 24, 48 and 72 h of incubation. Cells were colored with the Giemsa stain (Sigma-Aldrich Chemie GmbH; Taufkirchen, Germany). Briefly, 100μL of pure ethanol was added to a 96 well plate containing the treated cells by first removing the nutrient medium. Cells were left in ethanol for five minutes, and then, after removing ethanol, 100 μL of the Giemsa stain was added. Plates remained for 15 min at room temperature. Finally, the stain was removed and cells were washed with 100 μL of NaCl 0.9% (*v/v*).

### 2.3. Data Analysis

Multiparameter analyses were performed with GraphPad prism 8.0 (GraphPad Software Inc.; San Diego, CA, USA). All the results were expressed as mean ± SD. Comparative analysis between multiple groups was performed by two-way ANOVA to calculate significance of the mean differences between groups. Nonlinear regressions were performed using the equation:

(2) $$Y=Bottom+\frac{Top-Bottom}{1+10^{X-\log{\mathrm{IC}}_{50}}},$$

IC_50_ curves were calculated by the equation:

(3) $$Fifty=\frac{Top+Baseline}{2Y}=\frac{Bottom+(Top-Bottom)}{1+10^{(\log{\mathrm{IC}}_{50}-X)\cdot HillSlope+\log\left(\frac{Top-Bottom}{Fifty\_Bottom-1}\right)}},$$

Additionally, the percentage of cell viability was calculated as follows:

(4) $$\frac{\mathrm{FL}{\left(\mathrm{nm}\right)}_{Cells\_Exposed\_to\_drug}-\mathrm{FL}{\left(\mathrm{nm}\right)}_{wells\_containing\_only\_medium}}{\mathrm{FL}{\left(\mathrm{nm}\right)}_{Cells\_Not\_Exposed\_to\_drug}-\mathrm{FL}{\left(\mathrm{nm}\right)}_{wells\_containing\_only\_medium}}\times 100$$

where FL is Fluorescence in nm.

The statistical significance was accepted as *p* ≤ 0.05.

## 3. Results

### 3.1. Phase Solubility Analysis

Ideally, for 1:1 drug/CD complex, a phase solubility profile should give a straight line with a slope less than unity and an intercept (*S_int_*) equal to the intrinsic solubility of the drug *S*_0_. Subsequently, the stability constant (*K*_1:1_) is calculated from the slope and *S*_0_ by the Higuchi–Connors equation [30]:

(5) $$K_{1:1}=\frac{Slope}{S_{0}\cdot (1-Slope)},$$

**Figure 2:**
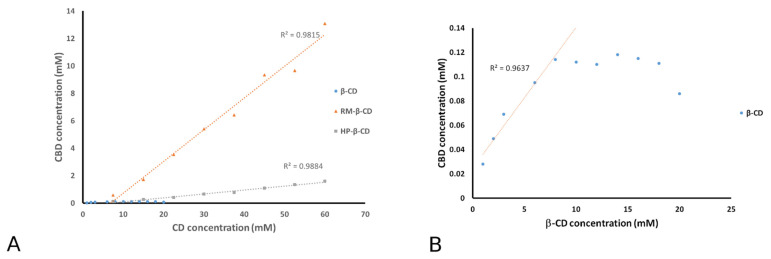
(**A**) Phase solubility diagrams of CBD/*β*-CD, CBD/RM-*β*-CD, and CBD/HP-*β*-CD systems in water at 25 °C (*n* = 3) and (**B**) the linear portion of the CBD/*β*-CD diagram.

In Figure 2A,B, the phase solubility profiles (i.e., plots of total CBD solubility, *S_t_*, in aqueous cyclodextrin solution, versus [*CD*]_t_) obtained for CBD/RM-*β*-CD, CBD/HP-*β*-CD, and CBD/*β*-CD inclusion complexes are illustrated.

Equation (5) illustrates that the determined *K*_1:1_ value is strongly affected by the accuracy of the intercept. Poorly soluble drugs (with intrinsic solubilities below about 0.1 mM) show negative intercept deviation, i.e., *S_int_* < *S*_0_, resulting in *A_L_*-type profiles in pure aqueous solutions [61]. CBD is a highly lipophilic drug; its intrinsic solubility in water (*S*_0_) has been measured at 0.2 μM by Mannila et al. [23]. The phase-solubility profiles of CBD/RM-*β*-CD and CBD/HP-*β*-CD (Figure 2A) are of the *A_L_*-type, giving negative *S_int_* values and thus resulting in negative *K*_1:1_ values, which is theoretically impossible. Therefore, the *K*_1:1_ values were determined by using the true intrinsic CBD solubility, *S*_0_ = 0.2 μM (Table 2).

In the case of the CBD/*β*-CD phase solubility profile (Figure 2B), the solubility of CBD increases linearly with increasing *β*-CD only in the range of 1–3 mM, indicating a typical *B*_s_-type profile. This profile type is not unusual for inclusion compounds of drugs in native *β*-CDs as the solubility limit of the drug/*β*-CD complex is reached within the concentration range of the *β*-CD. A *B*-type profile for CBD/*β*-CD has been also observed previously by Mannila et al. [23]. The *K*_1:1_ value in this case is estimated by the linear portion of the profile (Table 2).

According to all the aforementioned information, it is clear that the determination of *K*_1:1_ by the phase solubility method is significantly affected by the low CBD solubility. Therefore, it is more secure to use these results just to compare the affinity of CBD for native *β*-CD, RM-*β*-CD, and HP-*β*-CD. According to the values given in Table 2, the rank order of the CBD affinity with the examined CDs is: RM-*β*-CD > HP-*β*-CD > *β*-CD.

The Complexation Efficiency (CE) has been also estimated by the following equation:

(6) $$\mathrm{CE}=S_{0}K_{1:1}=\frac{Slope}{1-Slope},$$

CE is independent of both *S*_0_ and *S*_int_, as it is calculated solely from the slope of the phase solubility diagrams, and it expresses the concentration ratio between cyclodextrin in a complex and free cyclodextrin [62]. The results, summarized in Table 2, were used for the preparation of the solutions applied in the cell cytotoxicity experiments. Due to the low solubility of the CBD/*β*-CD inclusion complex observed in the phase solubility diagrams, an adequate CBD concentration in aqueous solution can be achieved only upon its complexation with RM-*β*-CD or HP-*β*-CD. Therefore, further investigation was focused on CBD inclusion complexes with methylated and hydroxypropylated *β*-CD hosts.

### 3.2. The Crystal Structure of CBD/DM-*β*-CD Inclusion Complex

**Figure 3:**
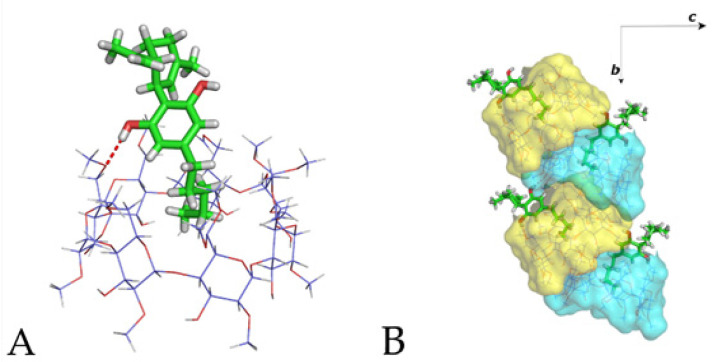
(**A**) The asymmetric unit of the CBD/DM-*β*-CD crystal structure and (**B**) the “herring bone” mode arrangement of the complex units along the *b*-axis.

The CBD/DM-*β*-CD crystallizes in the space group *P*2_1_2_1_2_1_ with the lattice parameters listed in Table 1. Its asymmetric unit contains one host (DM-*β*-CD) molecule and one guest (CBD) molecule partially entrapped in the host’s cavity; therefore, the host to guest stoichiometry of the inclusion complex in the crystalline state is 1:1. The guest is oriented with its aliphatic tail laying in the host’s cavity, whereas its limonene and benzenediol groups protrude from the primary rim of the host (Figure 3A). One hydroxyl of the benzenediol group of the guest is hydrogen bonded to a primary methoxy group of the encapsulant host. The other benzenediol hydroxyl of the guest is also hydrogen bonded to a hydroxyl of the secondary rim of a neighboring host. Adjacent host molecules are arranged in a “herring bone” mode along the crystallographic *b*-axis, with two primary methoxy groups of each host entering in the secondary rim of a neighboring host according to the self-inclusion tendency of DM-*β*-CD usually observed in the crystal structures of their inclusion complexes [31], whereas the interspace between them is filled by the protruding part of the guest molecule (Figure 3B). The crystal packing of the anhydrous complex units is completed by the parallel (along the *a*-axis) and antiparallel (along the c-axis) arrangement of these screw columns (based on the orientation of individual DM-*β*-CD units) via numerous C–H…O and H…H closed-shell interactions between the host molecules. Although 11 comparable unit cells were found in a CSD search, with cell matches based on differences between Krivy–Gruber reduced cells, no crystal structure of the DM-*β*-CD inclusion complex is amongst them. The “herring bone” arrangement of DM-*β*-CD complex units has been previously observed in several cases [31,63] due to the above mentioned self-inclusion tendency of the hosts. However, the lack of bridging water molecules in the case of the CBD/DM-*β*-CD crystal structure resulted in a unique crystal packing never observed before.

### 3.3. The Crystal Structure of CBD/TM-*β*-CD Inclusion Complex

**Figure 4:**
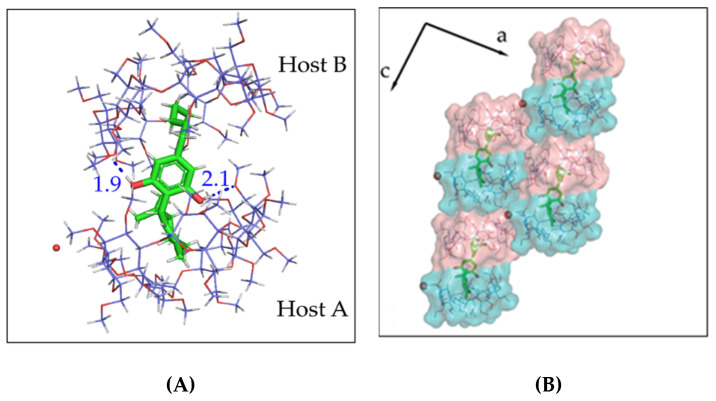
(**A**) The asymmetric unit of the CBD/TM-*β*-CD crystal structure. CBD is found encapsulated in an extended hydrophobic cavity formed by a TM-*β*-CD dimer. (**B**) The crystal packing of the CBD/TM-*β*-CD inclusion complex. Water molecules (red spheres) bridge adjacent dimers via hydrogen bonds.

The inclusion complex of CBD/TM-*β*-CD crystallizes in the monoclinic space group *P*2_1_. Unit cell parameters and further crystal lattice details are listed in Table 1. The asymmetric unit of the crystal structure consists of two hosts (hostA and hostB), one guest, and one water molecule. The guest is found encapsulated in the dimeric cavity formed by the two host molecules which are arranged in a head-to-head-type (the wide rim of hostA facing the wide rim of hostB) dimer; therefore, the host to guest stoichiometry of the inclusion complex in the crystalline state is 2:1 (Figure 4A). The mean plane of the glucosidic O4 *n* atoms of the hostA molecule is almost parallel to that of hostB (11.59°) and forms angle of 42.99° with the a*b* plane. It is worth mentioning that, although the formation of this kind of dimers is very common in native *β*-CD inclusion complexes, this is the first time that such a dimer has been observed for a TM-*β*-CD inclusion complex. The luck of hydroxyl groups in the TM-*β*-CD rims and the consequent absence of intermolecular hydrogen bonds between adjacent hosts do not favor the formation of TM-*β*-CD dimers. However, in the case of CBD/TM-*β*-CD, the encapsulation of the guest molecule in the extended hydrophobic dimeric cavity and the formation of hydrogen bonds between the benzenediol hydroxyls of the guest and the secondary methoxy groups of the hosts seem to tether the two TM-*β*-CD molecules in a head-to-head-type dimer. Τhe host molecules deviate significantly from a “round” annular structure, having an elliptical, cup-shaped conformation closed from the primary rim, which is a common feature in the majority of the TM-*β*-CD complexes due to the absence of intramolecular hydrogen bonds between the secondary methoxy groups of the host. The sole water molecule is located in the interspace of the complex units aiding the crystal packing by bridging adjacent complexes via hydrogen bonds with TM-*β*-CD molecules. The inclusion complex dimers are stacked in columns, which in turn are packed tightly via the aforementioned hydrogen bonds and numerous C–H…O and H…H closed-shell interactions between neighboring hosts (Figure 4B). The observed crystal packing is unique for cyclodextrin inclusion complexes. A search in CSD resulted in seven entries that share similar cell dimensions; however, none of them are relevant to cyclodextrin inclusion complexes.

### 3.4. Molecular Dynamics

The crystallographically determined atomic coordinates of CBD/DM-*β*-CD and CBD/TM-*β*-CD in addition to the 3 D model of CBD/HP-*β*-CD (1:1) generated by molecular docking predictions using the AutoDock Vina software were used as the initial structures for the MD simulations. The three distinct MD simulations were performed in an octahedral box of water for almost 20 ns at a pressure of 1 atm and temperature of 300 K and analyzed using CPPTRAJ implemented in AMBER 12, as described previously.

**Figure 5:**
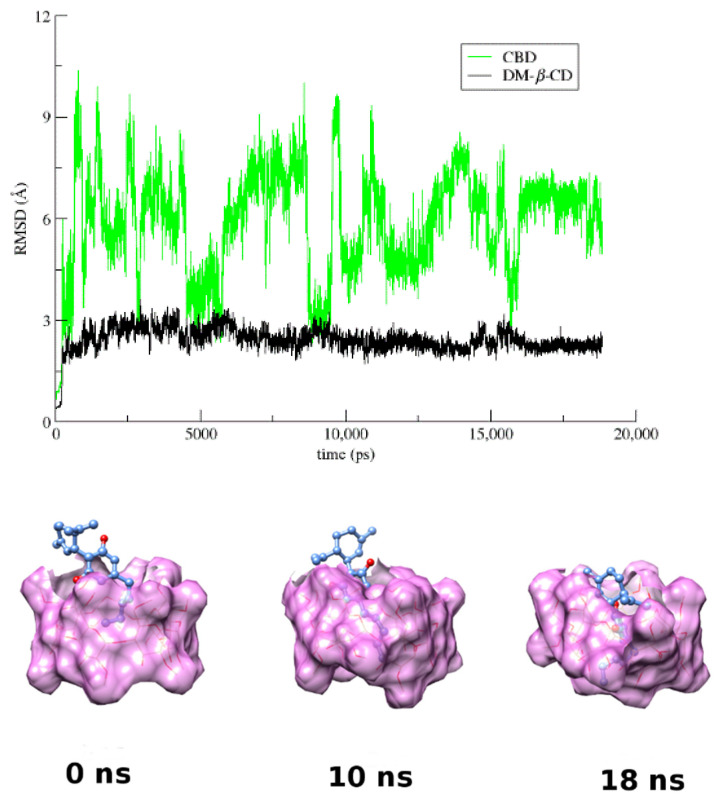
RMSD from the first frame of the trajectory for encapsulated CBD (green) in DM-*β*-CD (black) as a function of MD simulation time and representative snapshots of the CBD/DM-*β*-CD inclusion complex at 0, 10, and 18 ns.

In the case of the CBD/DM-*β*-CD inclusion complex, the 1:1 host to guest crystallographically determined structure was used as initial model. By monitoring the frames during simulation time-scale, it was observed that, in the absence of crystal contacts, the initially partial encapsulated guest CBD enters fully inside the hydrophobic DM-*β*-CD cavity maintaining its axial orientation (Figure 5). More specifically, the aliphatic tail of CBD penetrates the CD cavity from its primary rim and remains near the O4 n atoms plane for almost 14 ns. Subsequently, the guest is immersed deeper into the cavity with its benzenediol ring found near the O4 n plane (Figure 5). The RMSD plot (Figure 5) of the encapsulated CBD and host DM-*β*-CD reveals the high mobility of the guest in this inclusion complex. In most of the simulation time, both hydroxyls of the benzenediol group of CBD are hydrogen bonded with alternate primary methoxy groups of the host (Figure S1A,B, Supplementary Materials), contributing to the overall stability of the system. The rapid decrease in the host–guest COM distance at 14 ns and 16 ns (Figure S1C) indicates a deeper immersion of the guest inside the DM-*β*-CD cavity.

**Figure 6:**
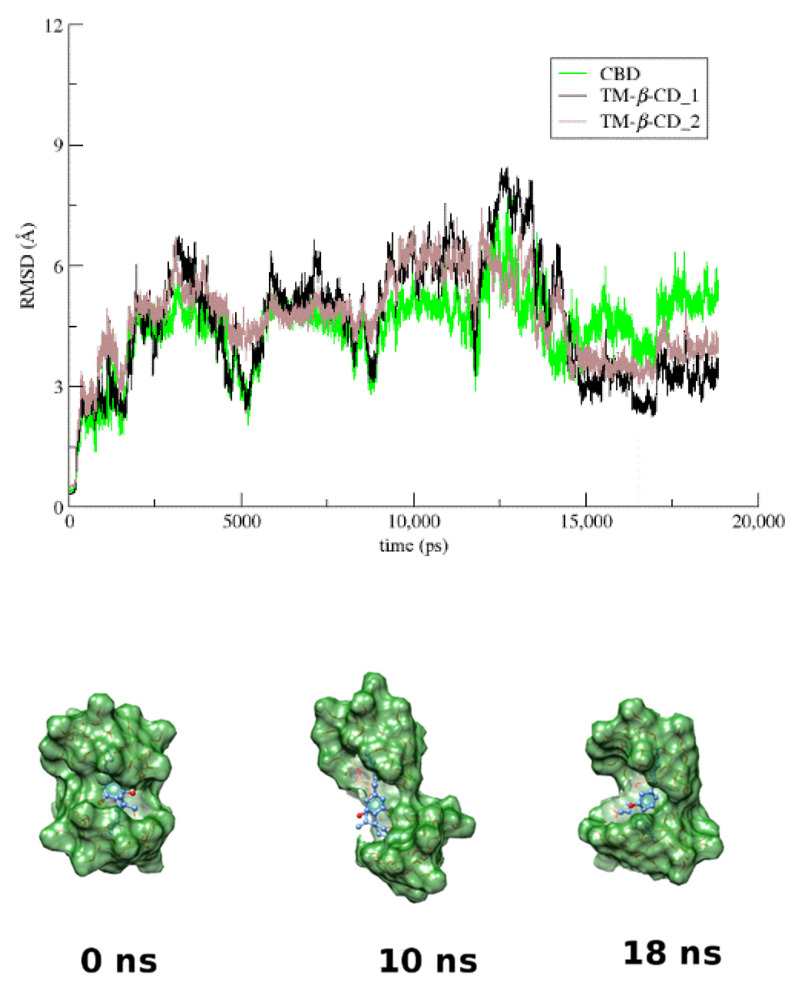
RMSD from the first frame of the trajectory for encapsulated CBD (green) in TM-*β*-CD dimer (black and brown) as a function of MD simulation time and representative snapshots of CBD/TM-*β*-CD inclusion complex at 0, 10, and 18 ns.

In the case of the CBD/TM-*β*-CD complex, the host to guest stoichiometry of the initial model is 2:1, as determined by the crystallographic studies. Although the dimer is severely distorted during the time frame of the simulation, the guest remains encapsulated in the dimeric cavity holding the two host molecules together (Figure 6). The RMSD plot of the guest and the hosts (Figure 6) shows that the mobility of the TM-*β*-CD molecules forming the dimer follows that of the encapsulated CBD. As the guest moves “up and down” (Figure S2E) retaining its orientation in the dimeric cavity, it is always tethered to the hosts, with hydrogen bonds formed between the hydroxyls of its benzenediol group and secondary methoxy groups of the hosts (Figure S3A–D).

**Figure 7:**
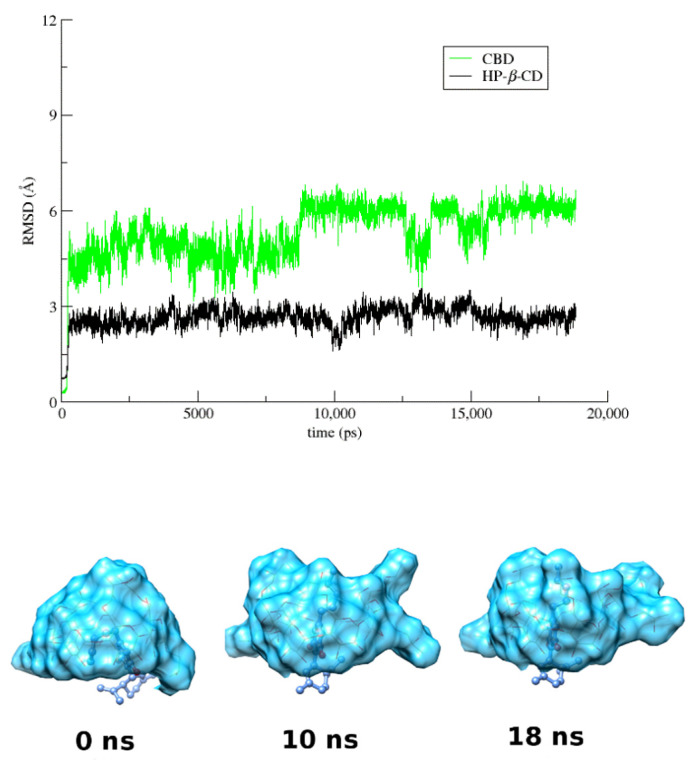
RMSD from the first frame of the trajectory for encapsulated CBD (green) in HP-*β*-CD (black) as a function of MD simulation time and representative snapshots of CBD/HP-*β*-CD inclusion complex at 0, 10, and 18 ns.

Finally, in the case of the CBD/HP-*β*-CD complex, whose 1:1 host to guest initial model was obtained by molecular docking, the guest CBD remains constantly encapsulated inside the hydrophobic HP-*β*-CD cavity, and in spite of the variation of its immersion depth into the cavity of the host, the entrapped molecule never changes its axial orientation (Figure 7 and Figure S3C). The hydroxyls of the benzenediol group of CBD are hydrogen bonded to the secondary rim of HP-*β*-CD (Figure S3A,B), whereas the aliphatic tail of CBD is found deeply immerged in the CD cavity, adopting either a bent or a linear conformation towards the primary rim of the host.

Binding affinities for all examined inclusion complexes were estimated by the Molecular Mechanics/Generalized Born Surface Area (MM/GBSA) method, and the values of the various terms are listed in Table 3. In every case, Van der Waals interactions are predominant. Comparison of the binding affinities of the inclusion complexes indicates that the calculated value Δ*G*_(GB)_ for the formed dimer in the case of CBD/TM-*β*-CD is significantly higher than those of the monomers (cases of CBD/DM-*β*-CD and CBD/HP-*β*-CD), as the second host molecule increases the Van der Waals interactions with CBD. On the other hand, MM/GBSA calculations show that HP-*β*-CD exhibits the lowest affinity for CBD, agreeing with the low stability constant and CE estimated by the phase solubility study (Table 3).

### 3.5. The Biological Effects of CBD

#### 3.5.1. The Effects on Glioblastoma Cells (A172)

##### The Vehicle-Dependent Effect on Glioblastoma Cells (A172)

The A172 cells were incubated with CBD in various concentrations and using various vehicles as described in Section 2. The first approach concerned the investigation of the vehicle’s effects on the in vitro model by treating cells with varying concentrations of vehicles, as those used for the CBD/CD complexes. In Figure S4, cell viability results, along with a detailed analysis on the subject, are presented. DMSO per se had no effect on cell viability (Figure S4A), whereas RM-*β*-CD (Figure S4B) and HP-*β*-CD (Figure S4C) had a significant cytotoxic effect at concentrations ≥0.036 and ≥0.018, respectively. However, as it will be depicted further on, the effect of the CBD/cyclodextrin complexes was more evident and additive.

##### The Dose-Dependent Effect of CBD and Its Inclusion Complexes with CDs on Glioblastoma Cells (A172)

**Figure 8:**
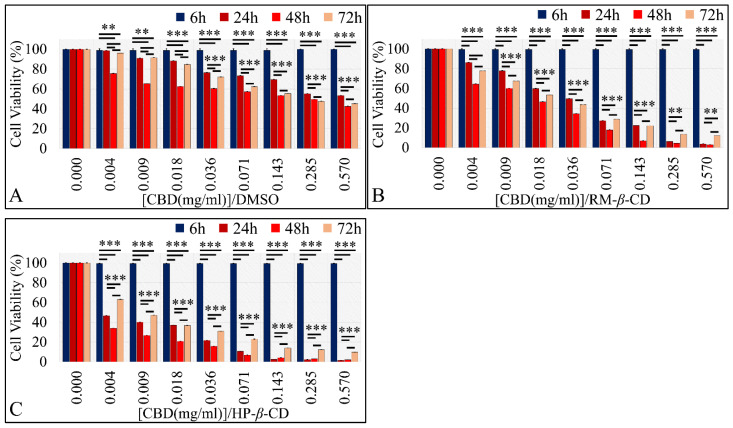
Dose-dependent effect of CBD and CBD inclusion complexes on A172 cells. (**A**) CBD in DMSO exhibited a significant dose-dependent effect for concentrations ≥ 0.009 mg/mL at 24 and 72 h and for all concentrations at 48 h; (**B**) CBD/RM-*β*-CD complex and (**C**) CBD/HP-*β*-CD manifested significant differences in a dose-dependent manner when compared with untreated cells ** depicts a significance at the *p* < 0.01 level and *** depicts a significance at the *p* < 0.0001 level. Each value represents the mean ± S.D. of triplicate experiments.

CBD and its inclusion complexes were indifferent at 6 h of exposure, as expected. Further on, CBD exhibited a dose-dependent effect at all time points; as its concentration increased, cell viability decreased. Significant differences were observed between controls (A172 cells without treatment, only staining agent) and 0.57, 0.285, 0.143, 0.071, 0.036, 0.018, and 0.009 mg/mL at 24 h and 72 h, whereas at 48 h CBD’s significant cytotoxic effect was observed for all concentrations (Figure 8A−C). When considering CBD–CDs inclusion complexes, CBD/RM-*β*-CD (Figure 8B) and CBD/HP-*β*-CD (Figure 8C) manifested significant differences in terms of cytotoxicity at all concentrations 0.004–0.57 mg/mL for all time points.

##### The Comparative Dose-Dependent Effect of CBD Complexes

The previous observations revealed that CBD’s activity was dependent upon the vehicle implemented. In particular, when comparing the drug effectiveness with respect to vehicle, HP-*β*-CD was the most effective combination in all tested concentrations and at all time points, i.e., 24 h (Figure S5A), 48 h (Figure S5C), and 72 h (Figure S5E). Significant differences between CBD and CBD/RM-*β*-CD, as well as CBD and CBD/HP-*β*-CD, were observed at all time points (24, 48, and 72 h) and all concentrations; CBD inclusion complexes manifested better cytotoxic performance as compared to CBD diluted in DMSO. HP-*β*-CD manifested better cytotoxic performance when compared to RM-*β*-CD at all time points (*p <* 0.0001 between CBD/HP-*β*-CD and CBD/RM-*β*-CD inclusion complexes for all concentrations and at all time points) (Figure S5A,C,E).

In order to better visualize the effect of all vehicles on cell viability, the latter was plotted with respect to the *log*_2_ of CBD concentration and the logarithmic regression of the observed cell viability. It was verified that the most effective CBD complex was CBD/HP-*β*-CD, followed by CBD/RM-*β*-CD, for all time points, i.e., 24 h (Figure S5B), 48 h (Figure D), and 72 h (Figure S5F). In addition, when regressing the cytotoxicity curves, cells recovered partly after 48 h of treatment. This was particularly evident for the CBD/RM-*β*-CD and CBD/HP-*β*-CD complexes.

##### The Time-Dependent Effect of CBD and Its Inclusion Complexes with CDs on Glioblastoma Cells (A172)

**Figure 9:**
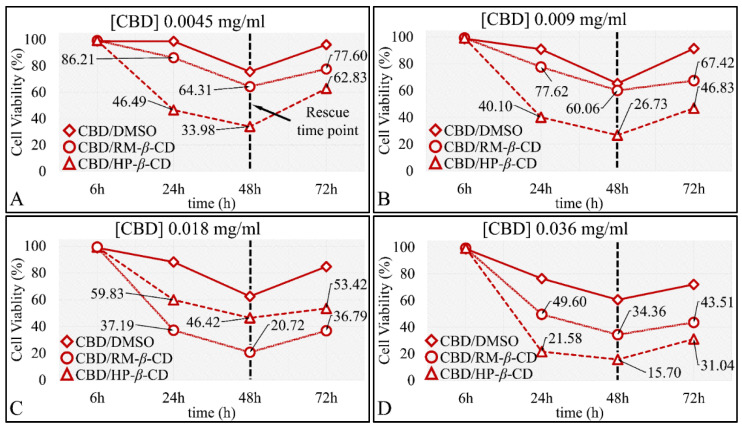

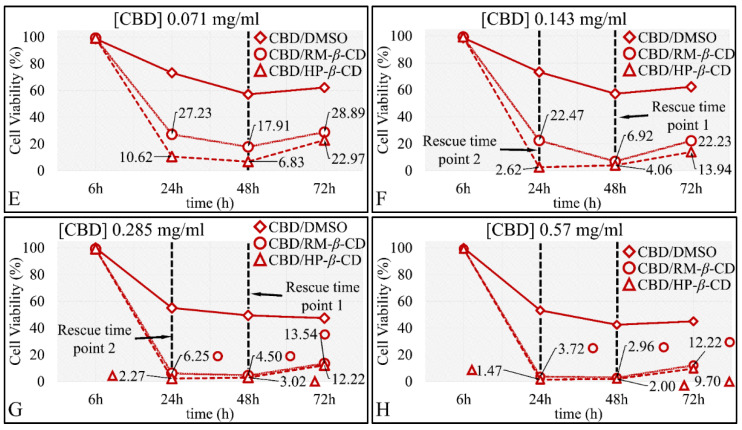
Comparative effectiveness of CBD and CBD inclusion complexes on A172 cells, with respect to time in particular, the concentrations 0.0045 mg/mL (**A**), 0.009 mg/mL (**B**), 0.018 mg/mL (**C**), 0.036 mg/mL (**D**), 0.071 mg/mL (**E**), 0.143 mg/mL (**F**), 0.285 mg/mL (**G**), and 0.57 mg/mL (**H**) are presented. The most effective inclusion complexes were the CBD/HP-*β*-CD and CBD/RM-*β*-CD in all concentrations between the 24 and 72 h cytotoxicity (*p* < 0.0001), followed by CBD/DMSO. Each value represents the mean ± SD of triplicate experiments.

Further on, the time-dependent efficacy of CBD and its inclusion complexes at each concentration tested was analyzed. In particular, measurements were performed at time points of 6 h, meaning at the time just after the addition of the drug and the staining agent and every 24 h thereafter, i.e., at 24 h, 48 h and 72 h. According to the examination of the time-dependent effect for each concentration tested at 6 h, as expected, no significant differences were present with respect to drug and vehicles. CBD was more cytotoxic at 48 h at all concentrations (*p* < 0.001 for 48 vs. 24 h and *p* < 0.001 for 48 vs. 72 h for all concentrations) (Figure 9), whereas at 72 h for all concentrations except of 0.28 mg/mL, there was gradual recovering of cells from the cytotoxicity of CBD at 48 h, with the effect being more prominent for concentrations ≤ 0.036 mg/mL. The same recovery effect was observed for both CD–CBD inclusion complexes for all concentrations (Figure 9).

All compounds at the 0.004 mg/mL concentration were more potent at 48 h (*p <* 0.001 for 24 vs. 48 h and *p <* 0.001 for 48 vs. 72 h for all concentrations). Interestingly, at 48 h a “Rescue Point” was observed, indicating that the cells were able to slightly recover after 48 h of exposure to any of the compounds (Figure 9A). When comparing 72 vs. 24 h, cell viability was still lower for cells exposed to CBD–DMSO and CBD/RM-*β*-CD, indicating a strong time-dependent effect (Figure 9A).

Almost the exact behavior was observed for the 0.009 and 0.036 mg/mL concentrations of CBD/DMSO or CBD/CDs. Cell viability was significantly lower at 48 h as compared to 24 h (*p <* 0.001), yet cell viability was higher at 72 h as compared to 48 h (*p <* 0.001), as cells also manifested a “Rescue Point”. When comparing 72 vs. 24 h, the time-dependent effect was observed for cells exposed to CBD/RM-*β*-CD (Figure 9B,D).

In the case of 0.018 mg/mL concentration, exposure for 48 h was the most effective for all three compounds. Cells were able to recover at 72 h from the cytotoxic effect observed at 48 h. When comparing 72 vs. 24 h, a significant cytotoxic effect (*p <* 0.001 for 72 vs. 24 h) on all compounds was observed (Figure 9C).

Similar to the previous observations, significant time-dependent cytotoxicity was observed at 0.071 mg/mL concentration between 48 vs. 24 h (*p <* 0.001) for all compounds. Cells exposed to CBD/DMSO and CD-inclusion complexes would recover in all cases at 72 h, as previously described. When comparing the 72 vs. 24 h, there was significant cytotoxicity (*p <* 0.001 for 72 vs. 24 h) for cells exposed to CBD/DMSO (Figure 9E).

Interestingly, although in the 0.143 mg/mL concentration the same pattern was manifested, a second “Rescue Point” appeared for the CBD/HP-*β*-CD complex after 24 h of exposure (Figure 9F). The exact same behavior was observed for cells exposed at concentrations of 0.285 mg/mL (Figure 9G) and 0.57 mg/mL (Figure 9H). In these concentrations, cells recovered after 24 h of exposure.

The phenomenon concerning the recovery behavior of cells when exposed to CBD inclusion complexes was further studied to reveal the velocity of recovery by using a simple calculation of the first derivative between two points (see Figure S6 for details of the calculation). Calculations between the end-points of 72 and 24 h were performed for all concentrations and all CBD inclusion complexes. A positive value indicated that cell viability at 72 h was higher as compared to 24 h, and a negative value was compatible with lower cell viability at 72 h as compared to 24 h. Thus, the lower (i.e., “the more negative”) the first derivative, the higher the effectiveness of the compound at the specified concentration. Hence, a high first derivative indicated that the compound was less effective or that cells succeeded to recover (dose-dependent nature of the compound for each concentration).

Additionally, as the derivative approached to zero, the compound exerted its effect at the first time points of exposure. On the contrary, as a derivative diverged from zero to negative values, the compound required more time in order to exert its effects (time-dependent nature of the compound at each concentration).

Interestingly, CBD/DMSO was the compound with the most prevalent time-dependent effects (Figure S6A), whereas CBD/HP-*β*-CD manifested a prevalent dose-dependent effect (Figure S6C). The most interesting behavior was presented by CBD/RM-*β*-CD. This complex exerted an almost perfect linear behavior with respect to the first derivative of cell viability, indicating that the compound interchanged its behavior between dose- and time-dependent with respect to its concentration. This interesting behavior indicates that the compound reaches a saturation level, above which its concentration is indifferent (Figure S6C).

Further on, CBD/DMSO (Figure S6A) manifested the lowest number of concentrations of cell recovery, while CBD/RM-*β*-CD (Figure S6B) manifested a linear behavior with respect to the effects of CBD. In the case of CBD/RM-*β*-CD, a dose-dependent behavior was observed for concentrations between 0.004 and 0.071 mg/mL (Figure S6B). Concerning CBD/HP-*β*-CD (Figure S6C), despite its most apparent cytotoxicity, cells were recovering fast at all concentrations, when compared to other CBD inclusion complexes.

##### IC_50_ of CBD Inclusion Complexes

**Figure 10:**
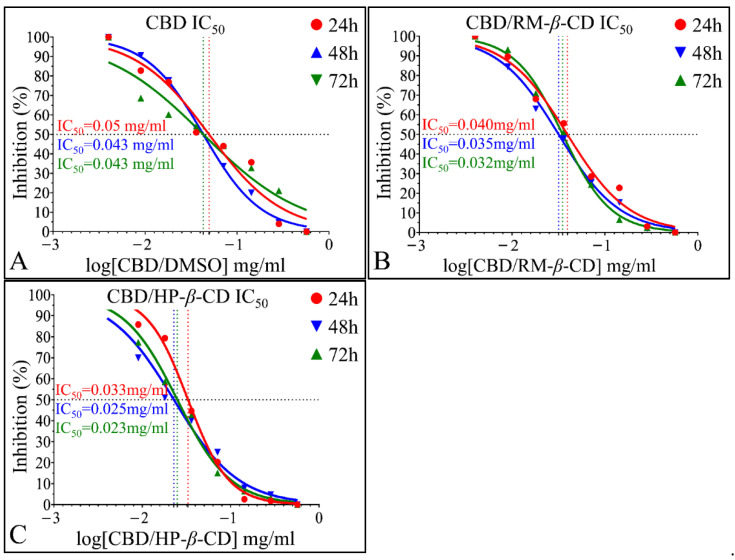
Calculation of IC_50_ of CBD and its inclusion complexes on A172 cells. (**A**) IC_50_ values were calculated to be 0.049 (*R*^2^ *=* 0.96) at 24 h, 0.043 mg/mL at 48 h, and 0.043 at 72 h for the sole CBD in DMSO; (**B**) the IC_50_ for the CBD/RM-*β*-CD inclusion complex was 0.040 mg/mL at 24 h, 0.035 mg/mL at 48 h, and 0.032 mg/mL at 72 h; (**C**) the IC_50_ for the CBD/HP-*β*-CD inclusion complex was 0.033 mg/mL at 24 h, 0.025 mg/mL at 48 h, and 0.023 mg/mL at 72 h.

The time-dependent effect of CBD and its inclusion complexes and the superior in vitro activity on A172 cells of CBD/HP-*β*-CD and CBD/RM-*β*-CD were also depicted via IC_50_ calculations. CBD/DMSO manifested an IC_50_ of 0.049 mg/mL at 24 h (*R*^2^ = 0.96), 0.043 mg/mL at 48 h (*R*^2^ = 0.93), and 0.043 mg/mL at 72 h (*R*^2^ *=* 0.99) (Figure 10A). Further on, IC_50_ for the CBD/RM-*β*-CD inclusion complex was calculated as 0.04 mg/mL, (*R*^2^*=* 0.99), 0.035 mg/mL (*R*^2^ *=* 0.99), and 0.032 mg/mL (*R*^2^ *=* 0.99) at 24, 48, and 72 h, respectively (Figure 10B). Finally, the IC_50_ for the CBD/HP-*β*-CD inclusion complex was found to be 0.033 mg/mL (*R*^2^ = 0.99), 0.025 mg/mL (*R*^2^ *=* 0.99), and 0.023 mg/mL (*R*^2^ *=* 0.97) at 24, 48, and 72 h, respectively (Figure 10C). The differences in IC_50_ calculations manifested a descending pattern both time-dependent and inclusion-dependent.

##### Microscopy Examination of CBD Effects on A172 Cells

A172 cells were also examined microscopically, and the indicative microscopy results under treatment with CBD/DMSO (Figure S7A), CBD/RM-*β*-CD (Figure S7B), and CBD/HP-*β*-CD (Figure S7C) are presented. Microscopy confirmed the aforementioned results of cell viability for the IC_50_ and highest concentrations.

##### Comparing the Effects of Vehicles and Vehicle/CBD Complexes

**Figure 11:**
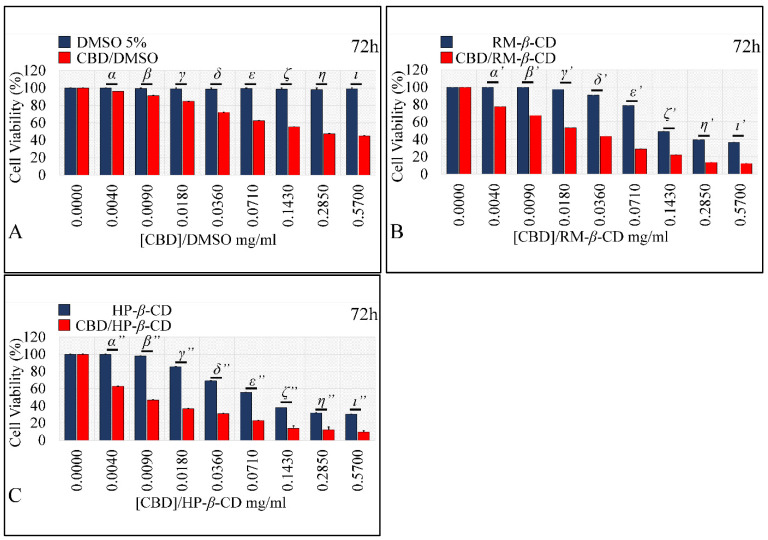
Comparison of vehicle-only and vehicle/CBD complexes on A172 cells at 72 h. (**A**) DMSO did not have any effect on cells, whereas CBD/DMSO manifested significant cytotoxicity as compared to the vehicle at all concentrations, except for the 0.004 mg/mL concentration, and at 72 h. (**B**) RM-*β*-CD. (**C**) HP-*β*-CD had a cytotoxic effect, yet the inclusion complexes manifested significantly higher cytotoxicity at all concentrations. The Greek letters above each bars-pair indicate the obtained p-value. In particular, *p*-values for the DMSO, CBD/DMSO comparison were: *α* = 0.07, *β* = 0.02, *γ* = 0.03, *δ* = 0.02, *ε* = 0.009, *ζ* = 0.018, *η* = 0.02, *ι* = 0.013. The *p*-values for the RM-*β*-CD, CBD/RM-*β*-CD comparison were: *α’* = 0.0003, **β*’* = 0.005, *γ’* = 0.018, *δ’* = 0.0012, *ε’* = 0.0019, *ζ’* = 0.007, *η’* = 0.0017, *ι’* = 0.007. The *p*-values for the HP-*β*-CD, CBD/HP-*β*-CD comparison were: *α’’* = 0.002, **β*’’* = 0.002, *γ’’* = 0.003, *δ’’* = 0.0009, *ε’’* = 0.007, *ζ’’* = 0.01, *η’’* = 0.017, *ι’’* = 0.01.

As aforementioned, isolated CDs had a significant cytotoxic effect on glioblastoma cells. Thus, it was important to compare the effect of vehicle-only complexes vs. the effect of CBD-inclusion complexes in order to examine a possible additive effect on cell proliferation of CBD. DMSO did not exert any effect on cells, whereas the CBD/DMSO solution manifested at all concentrations significant cytotoxicity, as compared to the vehicle (Figure 11A), except for the lowest concentration of 0.004 mg/mL at 72 h. On the other hand, RM-*β*-CD had a cytotoxic effect by itself, yet the CBD/RM-*β*-CD complex manifested significantly higher cytotoxicity (Figure 11B) at all concentrations. Thus, it appeared that CBD had an additive effect on the already inhibitory effect of RM-*β*-CD. Finally, HP-*β*-CD also had a cytotoxic effect by itself, whereas the CBD/HP-*β*-CD complex manifested significantly higher cytotoxicity (Figure 11C) at all concentrations. As in the case of RM-*β*-CD, HP-*β*-CD behaved similarly, where it appeared that CBD had an additive effect on the already inhibitory effect of HP-*β*-CD.

#### 3.5.2. The Effects on Rhabdomyosarcoma Cells (TE671)

##### The Vehicle-Dependent Effect on Rhabdomyosarcoma Cells (TE671)

As in the case of the A172 cell, TE671 cells were incubated with varying concentrations of CBD’s vehicles, resembling those of the CBD/CD complexes, as described in Section 2. DMSO per se had no effect on cell viability (Figure S8A), whereas RM-*β*-CD (Figure S8B) and HP-*β*-CD (Figure S8C) exerted a significant cytotoxic effect at concentrations ≥0.036 and ≥0.009, respectively. Yet, as it will be shown further on, the effect of the CBD/cyclodextrin complexes was more evident and additive.

##### The Dose-Dependent Effect of CBD and its inclusion complexes with CDs on Rhabdomyosarcoma Cells (TE671)

**Figure 12:**
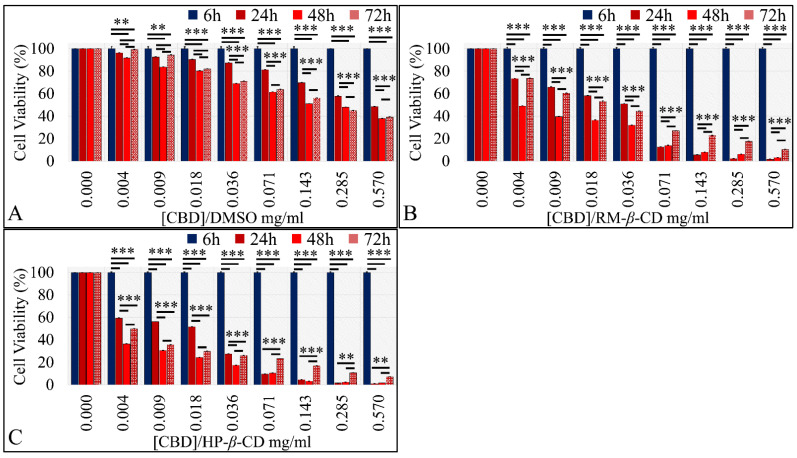
Dose-dependent effect of CBD and CBD inclusion complexes on TE671 cells. (**A**) CBD/DMSO exhibited a significant effect at all concentrations in a dose-dependent manner; (**B**) CBD/RM-*β*-CD complex; and (**C**) CBD/HP-*β*-CD demonstrated significant differences in a dose-dependent manner at all hours. ** depicts a significance at the *p <* 0.01 level and *** depicts a significance at the *p <* 0.0001 level. Each value represents the mean ± SD of triplicate experiments.

CBD and its inclusion complexes were indifferent at 6 h of exposure for the TE671 cells, as expected. CBD, irrespective of the vehicle, exhibited a dose-dependent effect at all time points. In particular, CBD/DMSO treatment manifested significant dose-dependent effects for all time points (*p* < 0.01); as its concentration increased, there was a reduction in viable cells (Figure 12A). The same dose-dependent effect was observed for CBD/RM-*β*-CD (*p* < 0.0001) and CBD/HP-*β*-CD (*p* < 0.0001) at all time-points, with the latter inclusion complex being more effective in terms of cytotoxicity (Figure 12B,C).

##### The Comparative Dose-Dependent Effect of CBD Complexes on Rhabdomyosarcoma Cells (TE671)

Upon comparison of the drug effectiveness with respect to vehicle, it appeared that HP-*β*-CD was the most effective combination in all tested concentrations and at all time points, i.e., 24 h (Figure S9A), 48 h (Figure S9C), and 72 h (Figure S9E). CBD inclusion complexes demonstrated superior cytotoxicity when compared to CBD/DMSO at all time points and all concentrations. Moreover, CBD/HP-*β*-CD was more efficient when compared to CBD/RM-*β*-CD at all time points (*p <* 0.01 between CBD/HP-*β*-CD vs. CBD/RM-*β*-CD for all concentrations and at all time points) (Figure S9A,C,E).

The contribution of vehicle in the cytotoxicity of the rhabdomyosarcoma cells was also visualized by plotting cell viability with respect to the *log*_2_ of the CBD concentration. In addition, the cytotoxicity curves were regressed. CBD diluted in DMSO manifested very similar behavior at 24 (Figure S9B), 48 (Figure S9D), and 72 h (Figure S9F). Finally, the most effective CBD/vehicle combination was the CBD/HP-*β*-CD, followed by CBD/RM-*β*-CD, for all time points, i.e., 24 (Figure S9B), 48 (Figure S9D), and 72 h (Figure S9F).

##### The Time-Dependent Effect of CBD and Its Inclusion Complexes with CDs on Rhabdomyosarcoma Cells (TE671)

**Figure 13:**
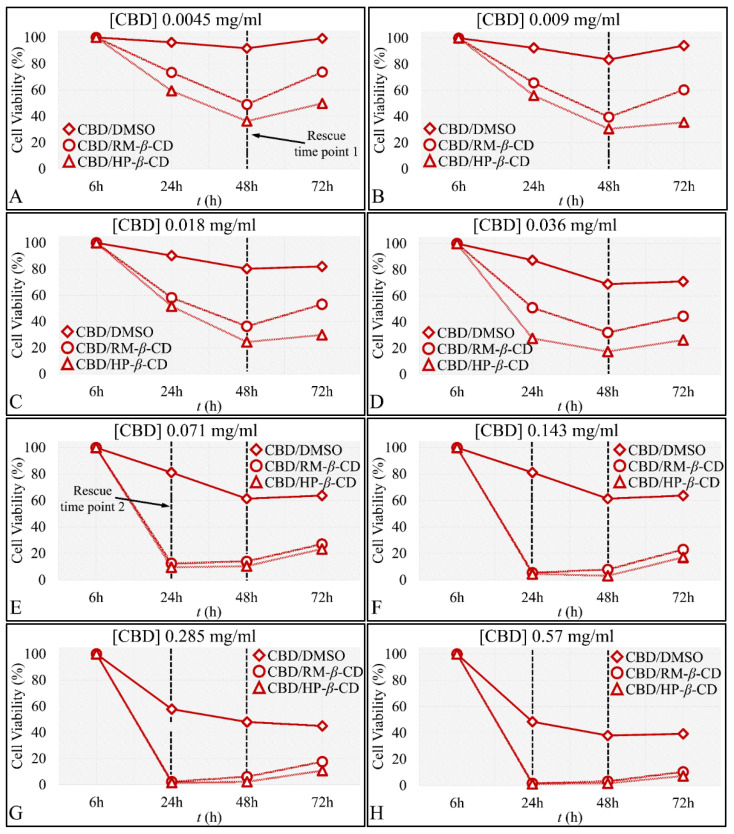
Comparative time-dependent effect of CBD and CBD inclusion complexes on TE671 cells. The 0.0045 mg/mL (**A**), 0.009 mg/mL (**B**), 0.018 mg/mL (**C**), 0.036 mg/mL (**D**), 0.071 mg/mL (**E**), 0.143 mg/mL (**F**), 0.285 mg/mL (**G**), and 0.57 mg/mL (**H**) concentrations are presented. The most effective inclusion complexes were the CBD/HP-*β*-CD and CBD/RM-*β*-CD for all concentrations, and between the 24 and 72 h cytotoxicity (*p <* 0.001), followed by CBD/DMSO. Each value represents the mean ± SD of triplicate experiments.

The time-dependent efficacy of CBD and its inclusion complexes on TE671 cells was further analyzed for each concentration. First, measurements were performed at time 6 h, meaning at the time just after the addition of the compound and the staining agent, and at 24, 48, and 72 h. At 6 h, as expected, no significant differences were present with respect to CBD and its vehicles. CBD was more cytotoxic at 48 h at all concentrations (*p <* 0.001 for 48 vs. 24 h, and *p <* 0.001 for 48 vs. 72 h for all concentrations) (Figure 13), whereas at 72 h and for all concentrations except for the 0.28 mg/mL, gradual recovering of cells from the effects of CBD at 48 h was observed (Figure 13).

For the 0.0045 mg/mL CBD concentration, cell viability when considering any of the three compounds was significantly lower at 48 h compared to 24 h (*p <* 0.001). However, cell viability was higher at 72 h compared to 48 h (*p <* 0.001), indicating a “Rescue Point” at 48 h, as cells were able to slightly recover after 72 h of treatment (Figure 13A). However, in comparison of 72 vs. 24 h, cell viability was lower for the CBD/HP-*β*-CD (*p <* 0.001) (Figure 13A).

Almost the exact behavior was observed for the 0.009 mg/mL CBD concentration, whereas cells manifested a “Rescue Point” at 48 h for all three compounds (*p <* 0.001 for 24 vs. 48 h and *p <* 0.001 for 48 vs. 72 h for all concentrations). Yet, when comparing 72 vs. 24 h, cell viability was lower for both inclusion complexes CBD/RM-*β*-CD and CBD/HP-*β*-CD (*p <* 0.001) (Figure 13B).

In the case of 0.018 mg/mL (Figure 13C) and 0.036 mg/mL (Figure 13D) concentrations, the same “Rescue Point” at 48 h appeared, indicating a repetition of the mechanism observed in the previous two lower concentrations. When comparing 72 vs. 24 h, a significant cytotoxicity was evident (*p* < 0.001 for 72 vs. 24 h for concentrations 0.018–0.036 mg/mL) for CBD/DMSO and both CBD/CDs inclusion complexes.

For the 0.071 mg/mL concentration, there was not a clear time-dependent effect of CBD/CDs, as cells demonstrated recovery at 48 and 72 h. However, in the case of CBD/DMSO, the compound was more efficient at 48 h, whereas cells recovered slightly at 72 h (Figure 13E).

Concerning the 0.143 mg/mL concentration, a time-dependent effect at 48 h for the CBD/DMSO and CBD/HP*-*β**-CD (*p* < 0.001 for 48 vs. 24 h) was evident. However, for all compounds, the “Rescue Point” was observed at 48 h, as cells recovered at 72 h (Figure 13F).

The same behavior was demonstrated by cells at the highest concentrations of 0.285 mg/mL (Figure 13G) and 0.57 mg/mL (Figure 13H). Cells recovered after 24 and 48 h when exposed to CBD/HP-*β*-CD or CBD/RM-*β*-CD. However, cell concentration at 24 h was bare minimum. A time-dependent effect was observed for the CBD/DMSO at 48 vs. 24 h and 72 vs. 24 h (*p* < 0.001 for 48 vs. 24 h and 72 vs. 24 h), whereas cells recovered at 48 h, following the usual pattern.

To further explore the recovery phenomenon of TE671, after CBD exposure, the first derivative between two points was used in order to estimate the possible differences in the “velocity” of recovery. Interestingly, CBD/DMSO (Figure S10A) manifested the lowest number of concentrations of cell recovery, whereas CBD/RM-*β*-CD (Figure S10B) demonstrated a threshold mechanism with respect to the effects of CBD. In the case of CBD/RM-*β*-CD, a dose-dependent behavior was observed for concentrations between 0.004 and 0.036 mg/mL (Figure S10B). On the other hand, in the case of CBD/HP-*β*-CD (Figure S10C), despite the most apparent cytotoxicity, the same threshold mechanism was observed, as cells at concentrations higher than 0.036 mg/mL started to recover. The observed behavior in the TE671 cells differed from the one of the A172 cells in the CBD/HP-*β*-CD complex.

##### IC_50_ of CBD Inclusion Complexes in the Rhabdomyosarcoma Cells (TE671)

**Figure 14:**
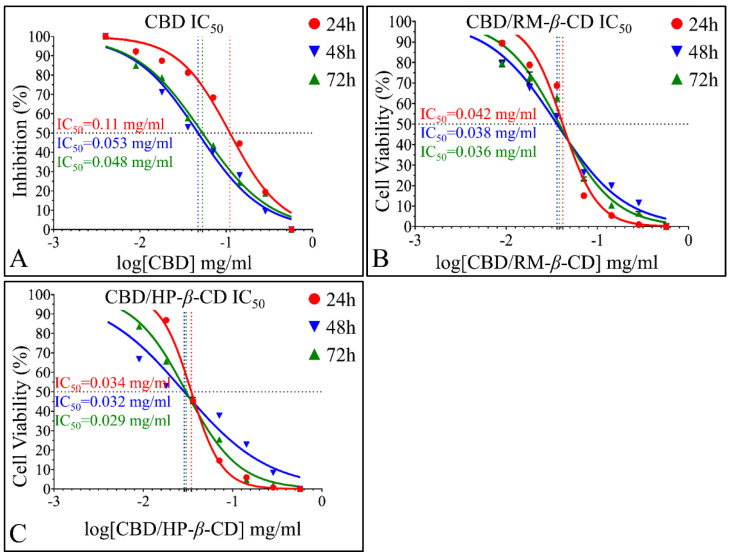
Calculation of IC_50_ of CBD and its inclusion complexes on TE671 cells. (**A**) IC_50_ values were calculated to be 0.11 mg/mL (*R*^2^ *=* 0.98) at 24 h, 0.053 mg/mL at 48 h (*R*^2^ *=* 0.98), and 0.048 mg/mL at 72 h (*R*^2^ *=* 0.98) for the CBD/DMSO inclusion complex. (**B**) The IC_50_ for the CBD/RM-*β*-CD inclusion complex was 0.042 mg/mL at 24 h (*R*^2^ *=* 0.97), 0.038 mg/mL at 48 h (*R*^2^ *=* 0.98), and 0.036 mg/mL at 72 h (*R*^2^ *=* 0.98). (**C**) The IC_50_ for the CBD/HP-*β*-CD inclusion complex was 0.034 mg/mL at 24 h (*R*^2^ *=* 0.99), 0.032 mg/mL at 48 h (*R*^2^ *=* 0.99), and 0.029 mg/mL at 72 h (*R*^2^ *=* 0.94).

The time-dependent effect of CBD and its inclusion complexes, as well the superior in vitro activity on TE671 cells of CBD/HP-*β*-CD and CBD/RM-*β*-CD, were verified via IC_50_ calculations. CBD/DMSO manifested an IC_50_ of 0.11 mg/mL at 24 h (*R*^2^ *=* 0.98) (the highest of all IC_50_ observed in all experiments), 0.053 mg/mL at 48 h (*R*^2^ *=* 0.98), and 0.048 mg/mL at 72 h (*R*^2^ *=* 0.98) (Figure 14A). IC_50_ for the CBD/RM-*β*-CD inclusion complex was found at 0.042 mg/mL (*R*^2^ *=* 0.97), 0.038 mg/mL (*R*^2^ *=* 0.98), and 0.036 mg/mL (*R*^2^ *=* 0.98) at 24, 48, and 72 h, respectively (Figure 14B). IC_50_ for the CBD/HP-*β*-CD inclusion complex was 0.034 mg/mL (*R*^2^ *=* 0.99), 0.032 mg/mL (*R*^2^ *=* 0.99), and 0.029 mg/mL (*R*^2^ *=* 0.94) at 24, 48, and 72 h, respectively (Figure 14C). The differences in IC_50_ calculations manifested a descending pattern both time-dependent and inclusion-dependent.

##### Microscopy Examination of CBD Effects on A172 Cells

TE671 cells were examined microscopically in order to validate the cytotoxicity results. Indicative microscopy images of TE671 cells under treatment with CBD/DMSO (Figure S11A), CBD/RM-*β*-CD (Figure S11B), and HP-*β*-CD (Figure S11C) are demonstrated. Microscopy confirmed the aforementioned results of cell viability for the IC_50_ and highest concentrations.

##### Comparing the Effects of Vehicles and Vehicle/CBD Complexes

**Figure 15:**
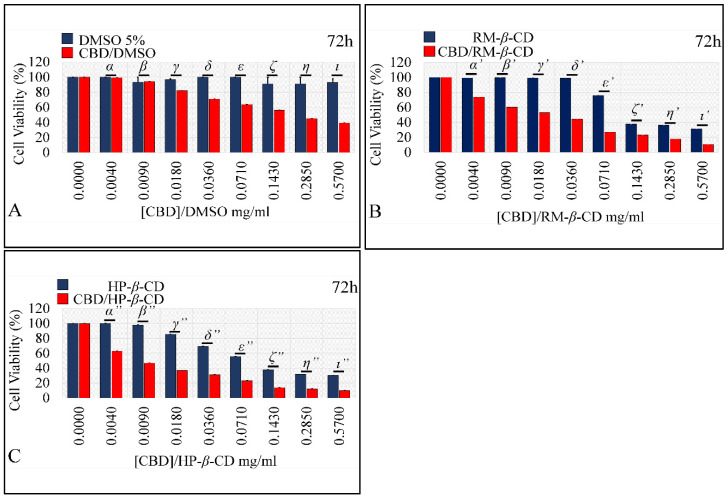
Comparison of vehicle-only and vehicle/CBD complexes on TE671 cells at 72 h. DMSO did not have any effect on cells, while the DMSO/CBD solution manifested significant cytotoxicity as compared to the vehicle (**A**) at all concentrations, except for the 0.004 and 0.009 concentrations. RM-*β*-CD (**B**) and HP-*β*-CD (**C**) had a cytotoxic effect, but their complexes manifested significantly higher cytotoxicity at all concentrations tested. The Greek letters above each bars-pair indicate the obtained p-value. In particular, *p*-values for the DMSO, CBD/DMSO comparison were: *α* = 0.03, *β* = 0.83, *γ* = 0.001, *δ* = 0.00001, *ε* = 0.000007, *ζ* = 0.018, *η* = 0.012, *ι* = 0.003. The *p*-values for the RM-*β*-CD, CBD/RM-*β*-CD comparison were: *α’* = 0.000007, **β*’* = 0.000005, *γ’* = 0.00007, *δ’* = 0.000012, *ε’* = 0.00004, *ζ’* = 0.0000001, *η’* = 0.0000017, *ι’* = 0.0000002. The *p*-values for the HP-*β*-CD, CBD/HP-*β*-CD comparison were: *α’’* = 0.000008, **β*’’* = 0.00004, *γ’’* = 0.00002, *δ’’* = 0.00002, *ε’’* = 0.00004, *ζ’’* = 0.00003, *η’’* = 0.00003, *ι’’* = 0.00008.

As aforementioned, cyclodextrin vehicles had a significant cytotoxic effect on rhabdomyosarcoma cells. Thus, the effect of isolated vehicles was compared to the effect of their respective CBD-inclusion complexes in order to examine whether CBD had an additive effect on cell cytotoxicity. DMSO did not exert any effect on cells, whereas the DMSO/CBD solution manifested significant cytotoxicity as compared to the vehicle (Figure 15A) at all concentrations, except for the 0.004 and 0.009 mg/mL concentrations, at 72 h. RM-*β*-CD had a cytotoxic effect by itself, yet the CBD/RM-*β*-CD complex manifested significantly higher cytotoxicity (Figure 15B) at all concentrations. HP-*β*-CD, also had a cytotoxic effect by itself, whereas the CBD/HP-*β*-CD complex demonstrated significantly superior cytotoxicity (Figure 15C) at all concentrations. The previous observations could be attributed to CBD’s additive effect, on the already inhibitory action of RM-*β*-CD, and HP-*β*-CD.

## 4. Discussion

### 4.1. CBD Inclusion Complexes

Τhe enhanced solubility of CBD upon complexation with native *β*-CD and its derivatives RM-*β*-CD and HP-*β*-CD was demonstrated by phase solubility study, revealing inclusion complexes of B_S_ type for CBD/*β*-CD and A_L_ type for CBD/RM-*β*-CD and CBD/HP-*β*-CD. The rank order of the CBD affinity with the examined CDs, in terms of the estimated CE and Kc values, is: RM-*β*-CD > HP-*β*-CD > *β*-CD. Due to the low solubility of CBD in presence of native *β*-CD, the investigation was focused on CBD inclusion complexes with methylated and hydroxypropylated *β*-CD hosts.

Single-crystal X-ray crystallography was used for crystal structure determination of the inclusion complexes of CBD in DM-*β*-CD and TM-*β*-CD. The CBD/DM-*β*-CD inclusion complex crystallizes in the *P*2_1_2_1_2_1_ space group, and its asymmetric unit contains one host and one partially entrapped CBD guest molecule revealing a 1:1 host to guest stoichiometry in the crystalline state. The CBD guest is found with its aliphatic tail entering the DM-*β*-CDs cavity from its primary rim, while the rest of the molecule is located mainly in the interspace formed by the adjacent neighboring hosts. The anhydrous complex units form screw channels deployed along the crystallographic *b*-axis in a “herring bone” fashion. The CBD/TM-*β*-CD inclusion complex crystallizes in the space group *P*2_1_. One guest molecule is accommodated inside a dimeric host cavity, hydrogen bonded with secondary methoxy groups of the hosts. Thus, the host to guest stoichiometry of the inclusion complex in the crystalline state is 2:1. The complexes stack along columns and the crystal packing consists of parallel and anti-parallel columns.

Furthermore, molecular dynamics simulations in explicit solvent, based on the crystallographically determined or docked models, and consequent calculations of the binding affinities of these inclusion complexes by the MM/GBSA method show the following:

The methylated *β*-CDs form stable inclusion complexes with CBD even in the absence of the crystal contacts. Especially in the case of the permethylated *β*-CD (TM-*β*-CD) host, which is susceptible to forming inclusion complexes by a pronounced induced fit mechanism, the crystallographically determined host dimer, rarely observed for TM-*β*-CD hosts, is found to firmly entrap the CBD guest with high binding affinity. A significant lower binding affinity was calculated in the case of CBD/HP-*β*-CD, agreeing with the experimental results of the phase solubility studies. From all the above, the conclusion drawn is that, among the examined CDs, the methylated *β*-CDs exhibit the best inclusion ability for CBD.

The understanding of the mode and structural details of inclusion of CBD in methylated-*β*CDs and HP-*β*-CD may be useful in the engineering of modified guest–host preparations with optimized pharmacological properties.

### 4.2. The Anti-Cancer Effects of CBD

Recurrent tumors remain particularly challenging, as little progress has been made towards improving patient outcomes and survival. Considering the broad spectrum of chemotherapeutics-induced side effects, additional pharmacological options for systemic cancer treatment are warranted. Cannabinoids are considered in cancer patients with refractory symptoms (nausea and vomiting, pain, depression, and anorexia) for their “palliative” effects, but with careful consideration of potential adverse reactions, and psychoactive side effects. Despite the lack of comprehensive clinical data, a large number of in vitro and in vivo studies suggest that cannabinoids elicit a broad array of anticancer effects on tumors of different origin, e.g., pheochromocytoma, glioma, neuroblastoma, rhabdomyosarcoma, leukemia, mantle cell lymphoma, non-small cell lung, skin, thyroid, prostate, breast, cervical, pancreatic, colon, gastric, bladder, head and neck squamous cell cancers [64,65,66,67,68,69,70,71,72,73,74]. Interestingly, there are no reports on the effects of CBD on rhabdomyosarcoma.

The interest in phytocannabinoids is part of increasing awareness of the medical potential of natural products with low toxicity. Considering CBD’s acceptable safety profile and its lack of psychoactivity, it is undoubtedly an important phytocannabinoid with a lot of reported pharmacological effects in several models of pathologies. It has been thoroughly tested in humans in controlled experimental studies and clinical trials for multiple sclerosis, schizophrenia, bipolar mania, social anxiety disorder, Huntington’s disease, Alzheimer disease and epilepsy. In children, CBD has been granted designation for treatment of intractable pediatric epilepsy syndromes, e.g., Dravet and Lennox–Gastaut syndrome, Fragile X, tuberous sclerosis, neonatal hypoxic ischemic encephalopathy, and pediatric schizophrenia [67,71,72,73,74].

Several molecular mechanisms have been proposed concerning CBD-mediated anticancer activity. CBD attenuates chronic inflammation by completely inhibiting the production of inflammatory cytokines by the tumor microenvironment, including IL-1*β*, IL-6, interferon-*β* (IFN-*β*), and TNF-α, all implicated in initiation and progression of several cancers. CBD selectively increases Natural Killer T-cells (NKT), which play an important role in cancer immune surveillance, as demonstrated by increased production of IL-12, a NK-stimulatory cytokine. An important action of CBD is the reduction in oxidative stress overload and lipid peroxidation in healthy cells, and at the same time the induction of a robust increase in intracellular ROS (Reactive Oxygen Species) with consequent mitochondrial damage, inhibition of cell survival, and self-renewal of cancer cells, sensitizing themto apoptosis. Additionally, CBD induces antiproliferative effects on tumor cells, mediated by a significant downregulation of PI3 K (Phosphoinositide 3-kinases)/Akt/mTOR (mammalian target of rapamycin) pathway, which promotes cell growth, proliferation, and survival and MAPK/ERK (extracellular signal-regulated kinases) pathway whose downstream targets are involved in the regulation of cell cycle entry and proliferation and the transcription of transcription factors, e.g., c-myc, CREB (cAMP response element-binding protein), and c-Fos. CBD induces autophagy and apoptosis-mediated cell death, mediated possibly by the antagonistic effect of CBD to GPR55 (G protein-coupled receptor 55) cannabinoid receptor, and subsequent recruitment of the death receptor, Fas/CD95, in lipid raft clusters. CBD apoptosis is also induced via TRPV1-mediated mitochondrial Ca^2+^ influx, CB2-mediated ceramide accumulation or induction of COX-2-dependent prostaglandins, and subsequent PPAR-γ (peroxisome proliferator-activated receptor gamma) accumulation in the nucleus. CBD modulates mitochondrial Ca^2+^ buffering and function (mitochondrial swelling, ROS production) and reduces the expression of mitochondrial-associated proteins prohibitin and STAT3 (signal transducer and activator of transcription 3) in cancer cells, thus intensifying cell stress and apoptosis. CBD inhibits cancer cell migration and invasion, via downregulating the expression of markers, associated with epithelial mesenchymal transition (EMT) and invasion, e.g., MT1–MMP (membrane type 1–matrix metalloproteinase 1), MMP-2, MMP9, PAI-1 (plasminogen activator inhibitor 1), FAK (focal adhesion kinase), Id-1 (Inhibitor of DNA binding-1), and inducing the expression of ICAM-1 and TIMP1 [64,65,66,67,68,69,70,71,72,73,74,75,76,77].

In A172 glioblastoma cell line, CBD exhibited a dose-dependent effect at 48 h and at 24 and 72 h for concentrations ≥ 0.009 mg/mL, whereas results were indifferent at 6 h of exposure, as expected. In the case of CBD–CDs inclusion complexes, CBD/RM-*β*-CD and CBD/HP-*β*-CD, a significant dose-dependent effect was observed at all-time points, starting from the lowest concentration of 0.004 mg/mL. When comparing the CBD vehicles’ effectiveness, the most effective combination in all tested concentrations and at all time points, in terms of cytotoxicity, appeared to be the CBD/HP-*β*-CD, followed by the CBD/RM-*β*-CD and the CBD/DMSO.

CBD’s cytotoxic effect was more prominent at 48 h and for all concentrations, except for the 0.28 mg/mL, as, at 72 h, cells partially recovered. The effect was more evident for concentrations ≤ 0.036 mg/m. However, when comparing 72 vs. 24 h, a time-dependent effect was observed for all concentrations, especially for concentrations ≥ 0.071 mg/mL. A similar recovery effect at 72 h was observed for both CD–CBD inclusion complexes for all concentrations, but cells did not recover to the pre-exposure levels.

In the TE671 cell line, CBD/DMSO exhibited a dose-dependent effect at all time points. The same effect was observed for CBD/RM-*β*-CD and CBD/HP-*β*-CD at all time-points, with the latter inclusion complex being more effective in terms of cytotoxicity.

Similar to the A172 cells, CBD was more effective at 48 h and for all concentrations, and a clear time-dependent action was depicted. At 72 h, cells partially recovered without reaching at any concentration the levels before treatment. When comparing 72 vs. 24 h, a time-dependent effect for concentrations ≥ 0.018 mg/mL was demonstrated. CBD inclusion complexes were more effective at 48 h for concentrations ≤ 0.036 mg/mL and ≤ 0.142 mg/mL, regarding CBD/RM-*β*-CD and CBD/HP-*β*-CD, respectively. When comparing 72 vs. 24 h, the time-dependent effect was evident for concentrations ≤ 0.036 mg/mL in the case of CBD/HP-*β*-CD and concentrations 0.009–0.036 mg/mL in the presence of CBD/RM-*β*-CD. The recovery effect at 72 h from the 48-h cytotoxicity was depicted for both CD–CBD inclusion complexes for all concentrations.

An important finding of the present research is that the two CD vehicles exerted cytotoxicity per se for concentrations ≥ 0.018 for HP-*β*-CD and ≥ 0.036 for RM-*β*-CD in the case of A172 cells and ≥0.009 for HP-*β*-CD and ≥0.036 for RM-*β*-CD. This has been previously reported, and it is known that CDs could be deployed, as therapeutic agents by themselves, without the use of any inclusion drugs, e.g., HP-*β*-CD for the treatment of Niemann–Pick Type C disease [78,79]. HP-*β*-CD possesses antitumor activity, inducing apoptosis on leukemic cells via disrupting cell membrane cholesterol homeostasis. However, the cytotoxicity of the inclusion complexes was not attributed solely to the action of the CDs; it appeared that CBD had an additive effect on the already inhibitory effect of the CDs. CDs probably increase the permeability of insoluble, hydrophobic drugs by making the drug available at the surface of the biological barrier, like the cell membrane, from where it partitions into the cell, without disrupting the lipid layers [80,81].

## 5. Conclusions

Phytocannabinoids have traditionally been used to alleviate cancer-related pain, to relieve chemotherapy related nausea, and stimulate appetite. However, in our study, we demonstrated a possible anti-proliferative effect of CBD on two tumor cell lines. In addition, the development of new CBD inclusion complexes with CDs is reported—among which HP-*β*-CD is FDA approved—in order to improve CBD’s properties [82]. CBD is highly lipophilic; thus, using a proper vehicle is a prerequisite for its proper delivery. CBD inclusion in CDs reduced its IC_50_ values in cytotoxicity experiments due to targeted, faster, and prolonged CBD release, leading to an increase in in vitro uptake by cancer cells. Similar results are expected in vivo, as CDs may aid CBD to bypass the blood–brain barrier and deliver CBD to the brain tissue in order to preferentially bind to the receptors overexpressed on the brain tumor cells. The inclusion of CBD and other phytocannabinoids in suitable CDs could provide a new promising approach for discovering new anticancer drugs, especially for cancer cells expressing relevant cannabinoid receptors, as CBD is considered a safe phytochemical, already tested in humans mainly for neurologic and psychiatric disorders.

## Figures and Tables

**Table 1:** Experimental details, crystal data, and refinement statistics.

	CBD/DM-*β*-CD	CBD/TM-*β*-CD
Crystal Data		
Chemical formula	C_56_ H_98_ O_35_·C_21_ H_30_ O_2_	2(C_63_ H_112_ O_35_)·C_21_ H_30_ O_2_·O
*M*_r_	1645.79	3189.49
Crystal system, space group	Orthorhombic, *P*2_1_2_1_2_1_	Monoclinic, *P*2_1_
Temperature (K)	100	100
*a*, *b*, *c* (Å)	15.5555 (18), 16.596 (2), 33.264 (4)	15.332 (3), 27.921 (6), 21.208 (7)
*b* (°)		110.932 (18)
*V* (Å^3^)	8587.6 (18)	8480 (4)
*Z*	4	2
Radiation type	Cu *K*α	Cu *K*α
μ (mm^−1^)	0.85	0.84
Crystal size (mm^3^)	0.4 × 0.2 × 0.08	0.4 × 0.3 × 0.2
Data collection		
Diffractometer	Bruker *APEX*-II	Bruker *APEX*-II
Absorption correction	Multi-scan *SADABS2016*/2—Bruker AXS area detector scaling and absorption correction	Multi-scan *SADABS2016*/2—Bruker AXS area detector scaling and absorption correction
*T*_min_, *T*_max_	0.61, 0.752	0.614, 0.753
No. of measured, independent, and observed [*I* > 2σ(*I*)] reflections	167917, 8422, 7132	151333, 24301, 17535
*R*_int_	0.083	0.087
ϑ_max_ (°)	49.1	60.2
(sin ϑ/λ)_max_ (Å^−1^)	0.490	0.563
Refinement		
*R*[*F*^2^ > 2σ(*F*^2^)], *wR*(*F*^2^), *S*	0.111, 0.308, 1.07	0.061, 0.136, 1.08
No. of reflections	8422	24301
No. of parameters	911	2011
No. of restraints	93	82
Δρ_max_, Δρ_min_ (e Å^−3^)	0.72, -0.73	0.35, -0.27

**Table 2:** Stability constants (K_1:1_) and Complexation Efficiency (CE) calculations of CBD inclusion complexes with the native and two modified *β*-CDs at 25 °C (*n* = 3).

Inclusion Complex	Type	Slope	K_1:1_ (M^−1^)	CE (%)
CBD/*β*-CD	B_s_	0.0116	58.7	1.2
CBD/HP-*β*-CD	A_L_^-^	0.0285	146.7	2.9
CBD/RM-*β*-CD	A_L_^-^	0.2312	1503.6	30.0

**Table 3:** Binding free energies and their standard deviations (kcal/mole) resulting from MM/GBSA analysis of the inclusion compounds of CBD/DM-*β*-CD (1:1), CBD/TM-*β*-CD dimer (2:1), and CBD/HP-*β*-CD (1:1).

	CBD/ DM-*β*-CD (1:1 Molar Ratio)	CBD/ TM-*β*-CD (1:2 Molar Ratio)	CBD/ HP-*β*-CD (1:1 Molar Ratio)
Δ*E*_vdW_	−31.84 ± 5.49	−47.89 ± 7.91	−30.30 ± 2.30
Δ*E*_ele_	−7.20 ± 3.20	−5.25 ± 2.93	−2.36 ± 3.02
Δ*E*_GB_	19.21 ± 5.00	29.24 ± 5.97	26.04 ± 3.85
Δ*E*_surf_	−3.95 ± 0.46	−5.95 ± 0.66	−3.83 ± 0.27
Δ*G*_gas_	−39.04 ± 7.54	−53.14 ± 9.62	−32.66 ± 3.63
Δ*G*_solv_	15.27 ± 4.64	23.29 ± 5.42	22.21 ± 3.77
Δ*G*(_GB_) ^a^	−23.78 ± 3.83	−29.85 ± 5.21	−10.45 ± 2.36
T⋅Δ*S*	−19.26 ± 1.73	−20.15 ± 4.53	−17.40 ± 1.49
Δ*G*_Bind_^b^	−4.52 ± 4.21	−9.70 ± 6.90	+6.95 ± 2.80

Δ*E*_vdW_ = Van der Waals contribution from molecular mechanics; Δ*E*_ele_ = electrostatic energy as calculated by the molecular mechanics force field; Δ*E*_GB_ = the electrostatic contribution to the solvation free energy, calculated by GB model; Δ*E*_surf_ = nonpolar contribution to the solvation free energy, calculated by an empirical model; ^a^ Δ*G*_(GB)_ = Δ*G*_solv_ + Δ*G*_gas_; ^b^ Δ*G*Bind = Δ*G*_(GB)_ + (T·Δ*S*).

## Data Availability

Crystallographic data have been deposited into the Cambridge Structural Database (CSD) under the deposition numbers CCDC: 2098419 and 2094890. Further data presented in this study are available on request from the corresponding author.

## Supplementary Materials

The following are available online at: https://www.mdpi.com/article/10.3390/pharmaceutics14040706/s1. Figure S1: (A,B) H-bond monitoring between CBD and host DM-*β*-CD (C) plot of host-guest Centre of Mass (COM) distance.; Figure S2: (A−D) H-bond monitoring between CBD and host TM-*β*-CD and (E) plots of hosts-guest COM distances; Figure S3: (A,B) H-bond monitoring between CBD and host HP-*β*-CD (C) plot of host-guest COM distance; Figure S4: Dose-dependent effect of vehicles on A172 cells; Figure S5: Comparative dose-dependent effect of CBD and CBD inclusion complexes on A172 cells; Figure S6: The comparative recovery velocity of CBD complexes; Figure S7: Microscopic examination of A172 cells exposed to CBD, and its inclusion complexes; Figure S8: Dose-dependent effect of vehicles on TE671 cells; Figure S9: Comparative dose-dependent effect of CBD and CBD inclusion complexes on TE671 cells; Figure S10: The comparative recovery velocity of CBD complexes in the TE671 cells; Figure S11: Microscopic examination of TE671 cells exposed to CBD, and its inclusion complexes.
