# Supplementary material for: Biophysical Studies and In Vitro Effects of Tumor Cell Lines of Cannabidiol and Its Cyclodextrin Inclusion Complexes
Source: Pharmaceutics. 2022 Mar 26;14(4):706. doi: 10.3390/pharmaceutics14040706 (PMC9027293; doi:10.3390/pharmaceutics14040706)
Supplement: Supplementary file 1 [file pharmaceutics-14-00706-s001.zip › pharmaceutics-1604483-SI.pdf]

# Supplementary Materials: Biophysical Studies and In Vitro Effects of Tumor Cell Lines of Cannabidiol and Its Cyclodextrin Inclusion Complexes

**Kyriaki Hatziagapiou, Kostas Bethanis, Eleni Koniari, Elias Christoforides, Olti Nikola, Athena Andreou, Aimilia Mantzou, George P. Chrousos, Christina Kanaka-Gantennbein and George I. Lambrou**

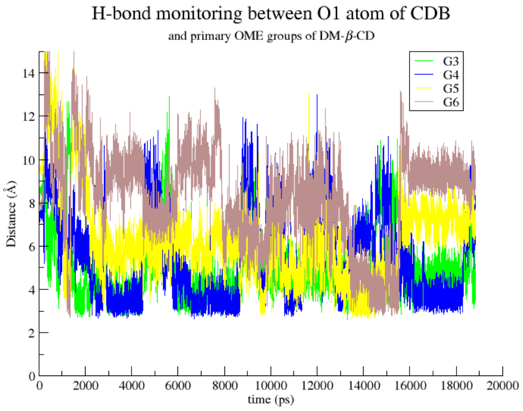


**(A)**

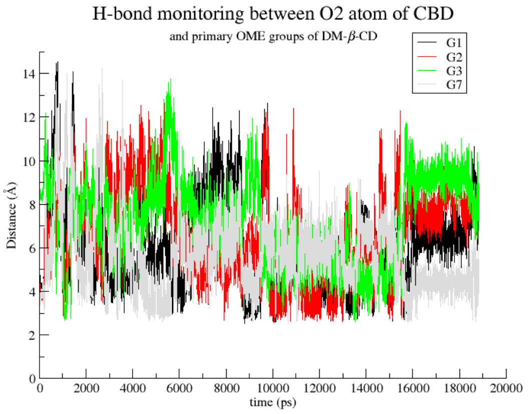


**(B)**

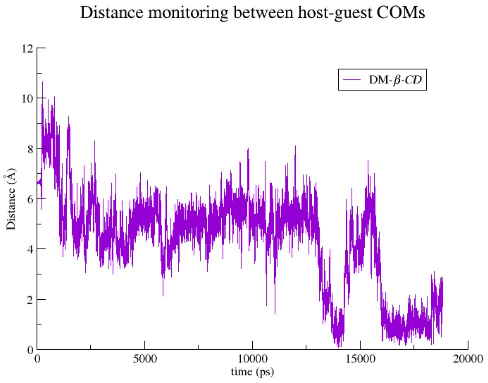


**(C)**

**Figure S1.** (**A**,**B**) H-bond monitoring between O1 and O2 atoms of CBD and primary methoxy groups (OME) of the host (glucose units G3, G4, G5 and G6) and (**C**) plot of host-guest Centre of Mass (COM) distance. Rapid decrease at 14 ns and 16 ns indicates the deeper immersion of the guest inside the DM-*β*-CD cavity.

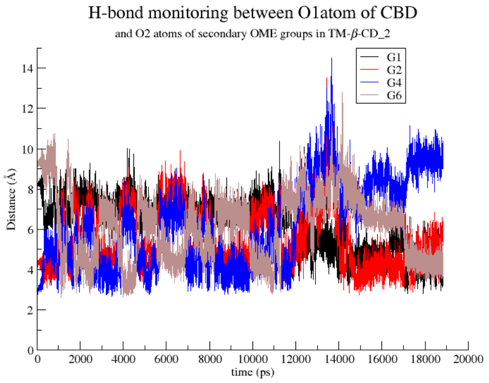


**(A)**

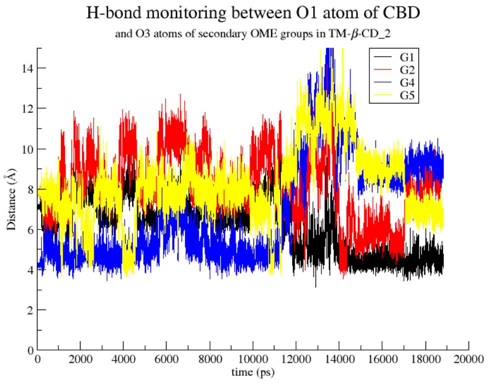


**(B)**

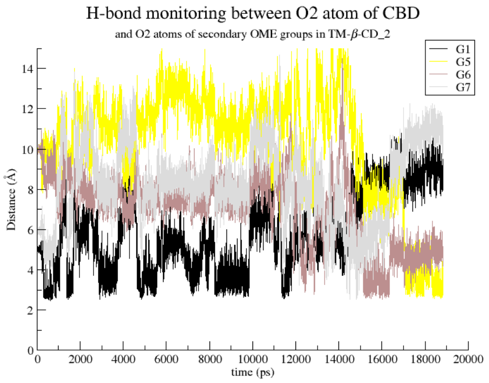


**(C)**

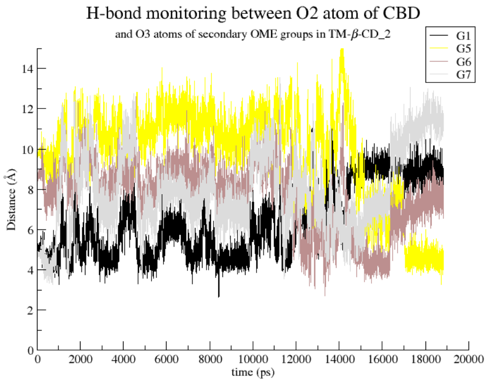


**(D)**

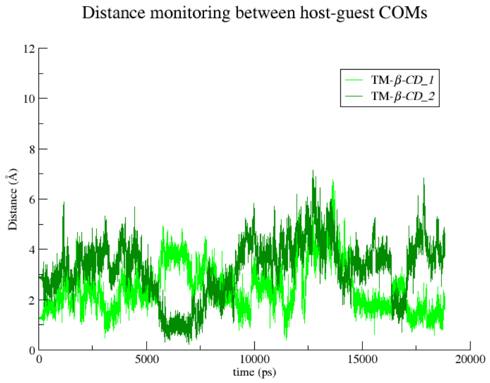


**(E)**

**Figure S2.** (**A–E**). H-bond monitoring between the hydroxyls of the benzenediol group of the guest and the secondary methoxy groups (OME) of the hosts and (**E**) plots of hosts-guest Centre of Mass (COM) distances.

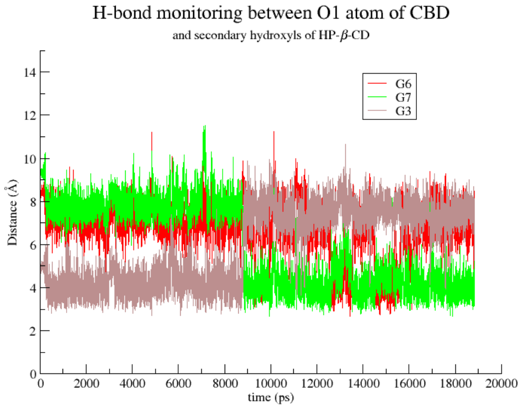


**(A)**

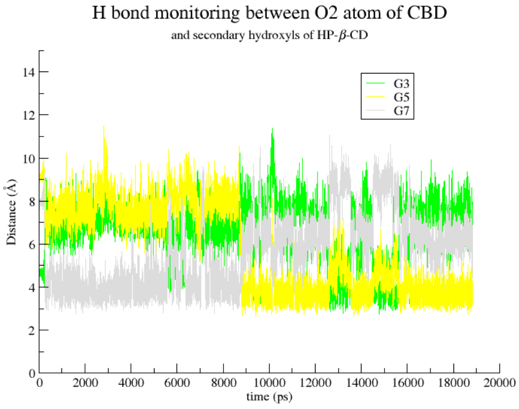


**(B)**

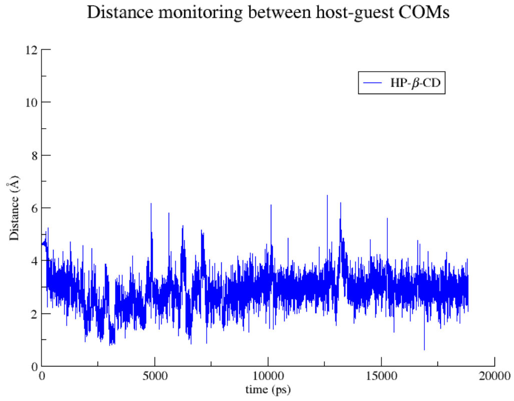


**(C)**

**Figure S3.** (**A**,**B**) H-bond monitoring between the hydroxyls of the benzenediol group of the guest and the secondary hydroxyl groups of the host and (**C**) plot of host-guest Centre of Mass (COM) distance.

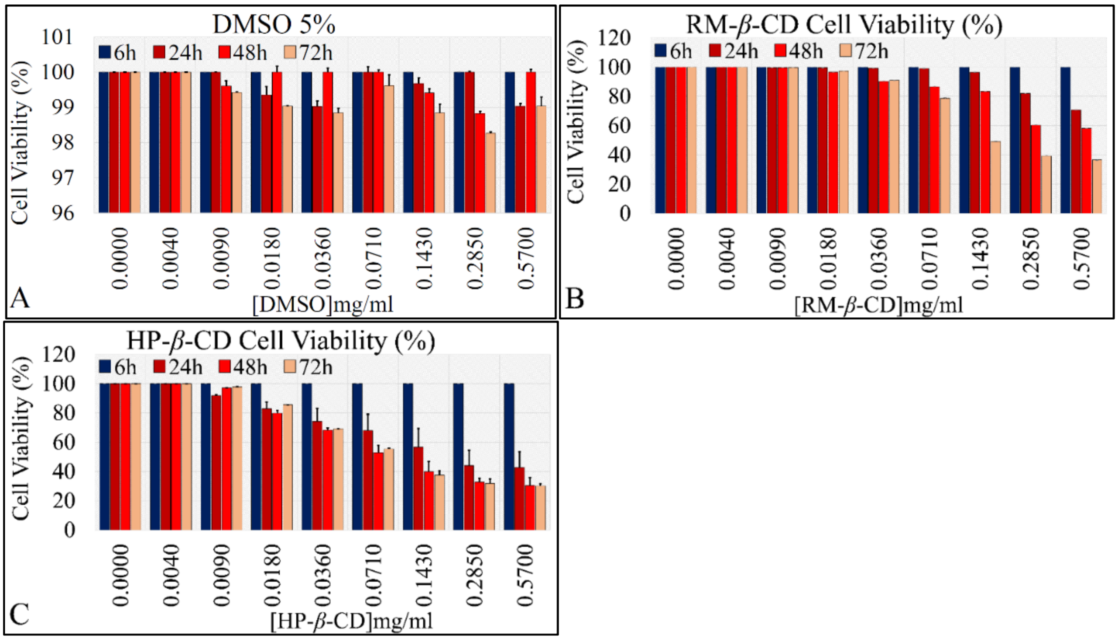


**Figure S4. Dose-dependent effect of vehicles on A172 cells.** Analyzing drug effects with respect to concentration for each time point studied (6, 24, 48, and 72 hours), revealed information in the role of the tested CD-vehicles. (**A**) DMSO treatment exhibited no significant cytotoxic effect as cell

viability did not fall below 97% for all concentrations and at all time points, (**B**) In the case of RM-$\beta$-CD and (**C**) HP-$\beta$-CD, cell viability was reduced in a dose- and time-dependent manner for concentrations ≥ 0.036, and ≥ 0.018, respectively. The two vehicles, manifested a cytotoxic effect by themselves, as they reduced cell viability down to approximately 30% for the higher concentrations. Note that no *p*-values are presented since the main focus was on the vehicle's effects.

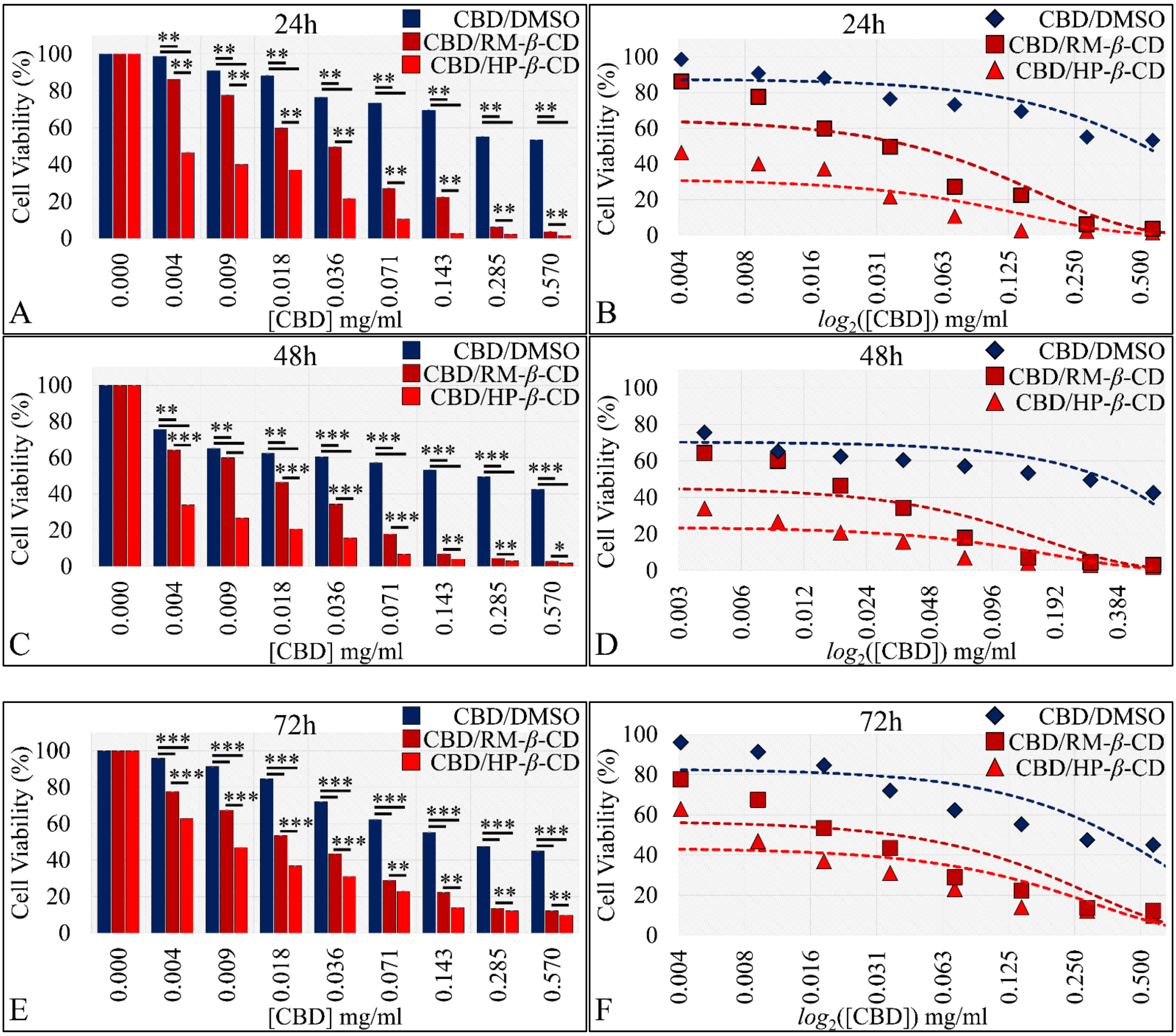


**Figure S5.** Comparative dose-dependent effect of CBD and CBD inclusion complexes on A172 cells. The comparative effectiveness of the inclusion complexes was investigated and presented together. The CBD/vehicle effectiveness appeared to follow the same pattern at all time points and in particular CBD/HP-$\beta$-CD was the most effective treatment, followed by CBD/RM-$\beta$-CD and CDB/DMSO. This effect was evident at all time points that is 24 hours (**A**,**B**), 48 hours (**C**,**D**) as well as 72 hours (**E**,**F**) * depicts a significance at the $p < 0.05$ level; ** depict a significance at the $p < 0.01$ level; *** depict a significance at the $p < 0.001$ level.

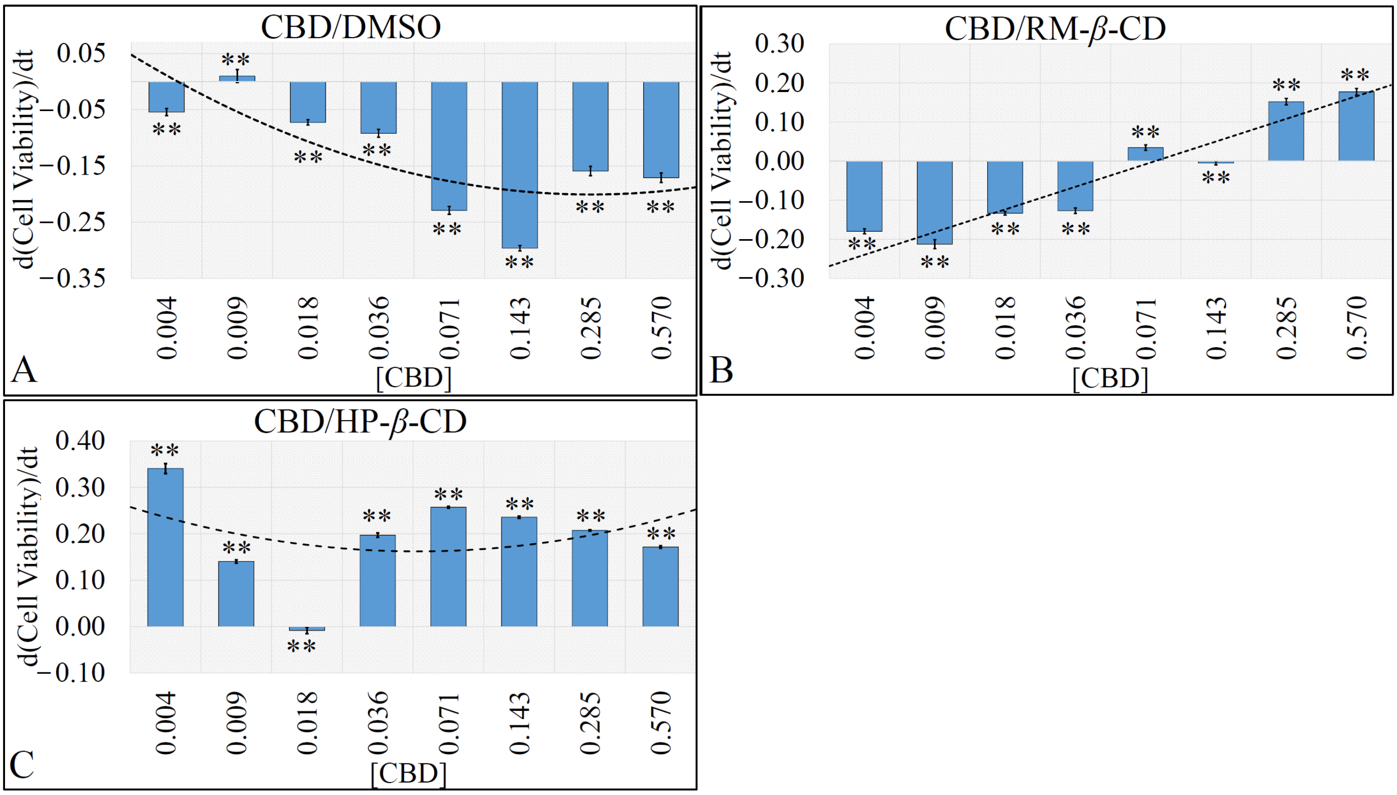


**Figure S6. The comparative recovery velocity of CBD complexes.** We calculated the "speed" by which cells recover after treatment, using the Equation (S1).

$$v_{Cell\_Recovery} = \frac{Cytotoxicity_{[CBD],t_2} - Cytotoxicity_{[CBD],t_1}}{t_2 - t_1} \qquad \text{(S1)}$$

where, $v$, is the calculated recovery speed at mg/ml/h. Calculations between the endpoint of 72 hours and 24 hours for all concentrations and all CBD inclusion complexes were performed. (**A**) Cells treated with CBD/DMSO did not recover alltogether and the observed differences were significant ($p < 0.001$). (**B**) Cells exposed to CBD/RM-$\beta$-CD for concentrations 0.071–0.57 mg/ml exchibited a cellular rescue, whereas for concentrations 0.004–0.036 mg/ml they did not demonstrate such behaviour. (**C**) Cells exposed to CBD/HP-$\beta$-CD manifested the "fastest" recovery rate. Yet, although the recovery rate under CBD/HP-$\beta$-CD treatment was high, its effectiveness still surpassed all other CBD inclusion complexes. The asterisks ** depict a significance at the $p < 0.01$ level. The asterisks above all bars indicate that the differences are significant among all concentrations and in all combinations.

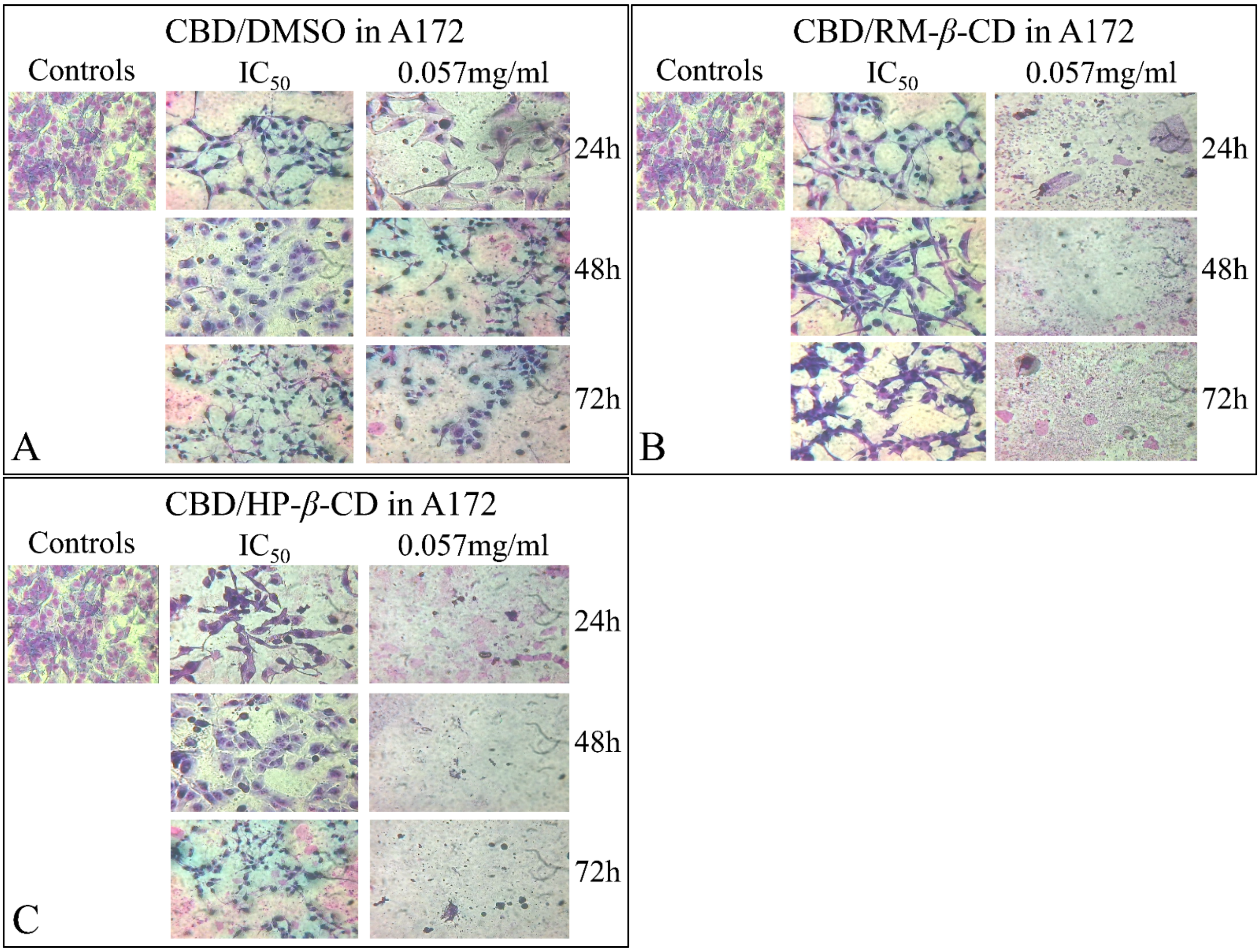


**Figure S7. Microscopic examination of A172 cells exposed to CBD, and its inclusion complexes.** Microscopy confirmed the cell viability when A172 were exposed to **(A)** CBD/DMSO, **(B)** CBD/RM-$\beta$-CD complex and **(C)** CBD/HP-$\beta$-CD complex.

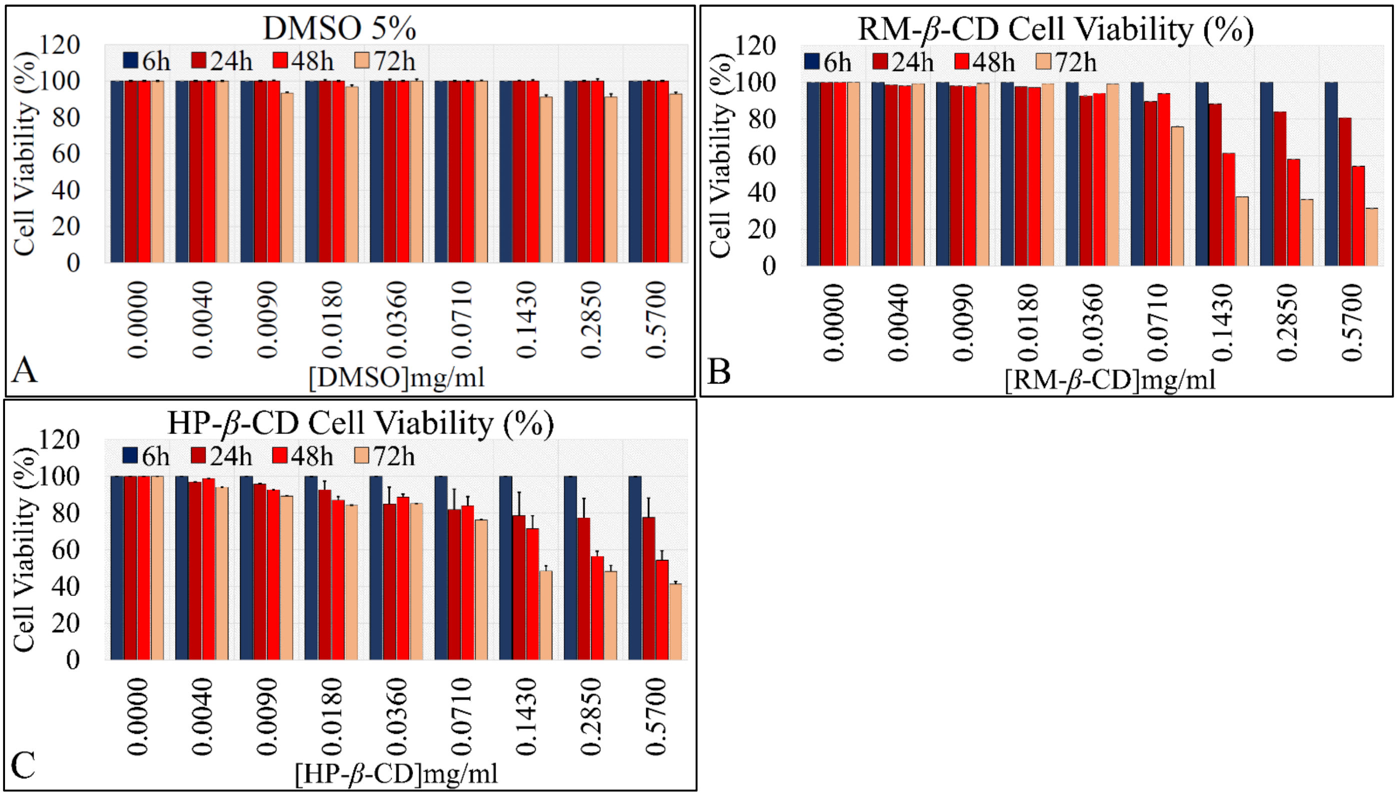


**Figure S8. Dose-dependent effect of vehicles on TE671 cells.** Analyzing drug effects with respect to concentration for each time point studied (6, 24, 48, and 72 hours), revealed information in the role of the tested CD-vehicles. **(A)** DMSO treatment exhibited no significant cytotoxic effect as cell viability did not fall below 90% for all concentrations and at all time points, **(B)** In the case of RM-$\beta$-CD and **(C)** HP-$\beta$-CD, cell viability was reduced in a dose- and time-dependent manner for concentrations ≥ 0.036, and ≥ 0.009, respectively. The two vehicles, manifested a cytotoxic effect by themselves, as they reduced cell viability down to approx. 30% for the higher concentrations. Note that no *p*-values are presented since the main focus was on the vehicle's effects.

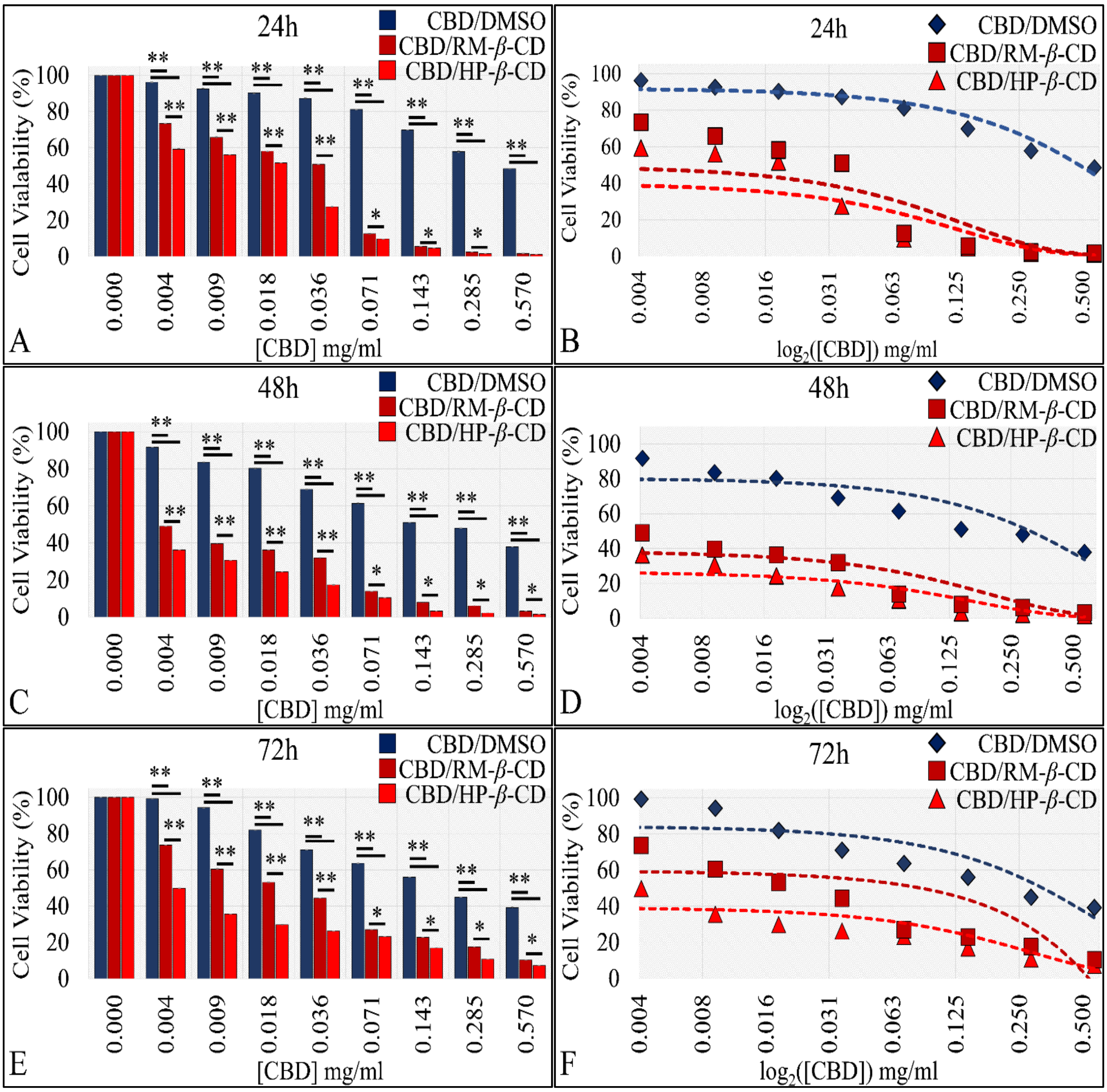


**Figure S9. Comparative dose-dependent effect of CBD and CBD inclusion complexes on TE671 cells**. The CBD/vehicle effectiveness appeared to follow the same pattern at all time points and in particular CBD/HP-*β*-CD was the most effective treatment, followed by CBD/RM-*β*-CD and CDB/DMSO. This effect was evident at all time points, i.e., 24 (**A**,**B**), 48 (**C**,**D**), and 72 hours (**E**,**F**); * depicts a significance at the $p < 0.05$ level; ** depict a significance at the $p < 0.01$ level.

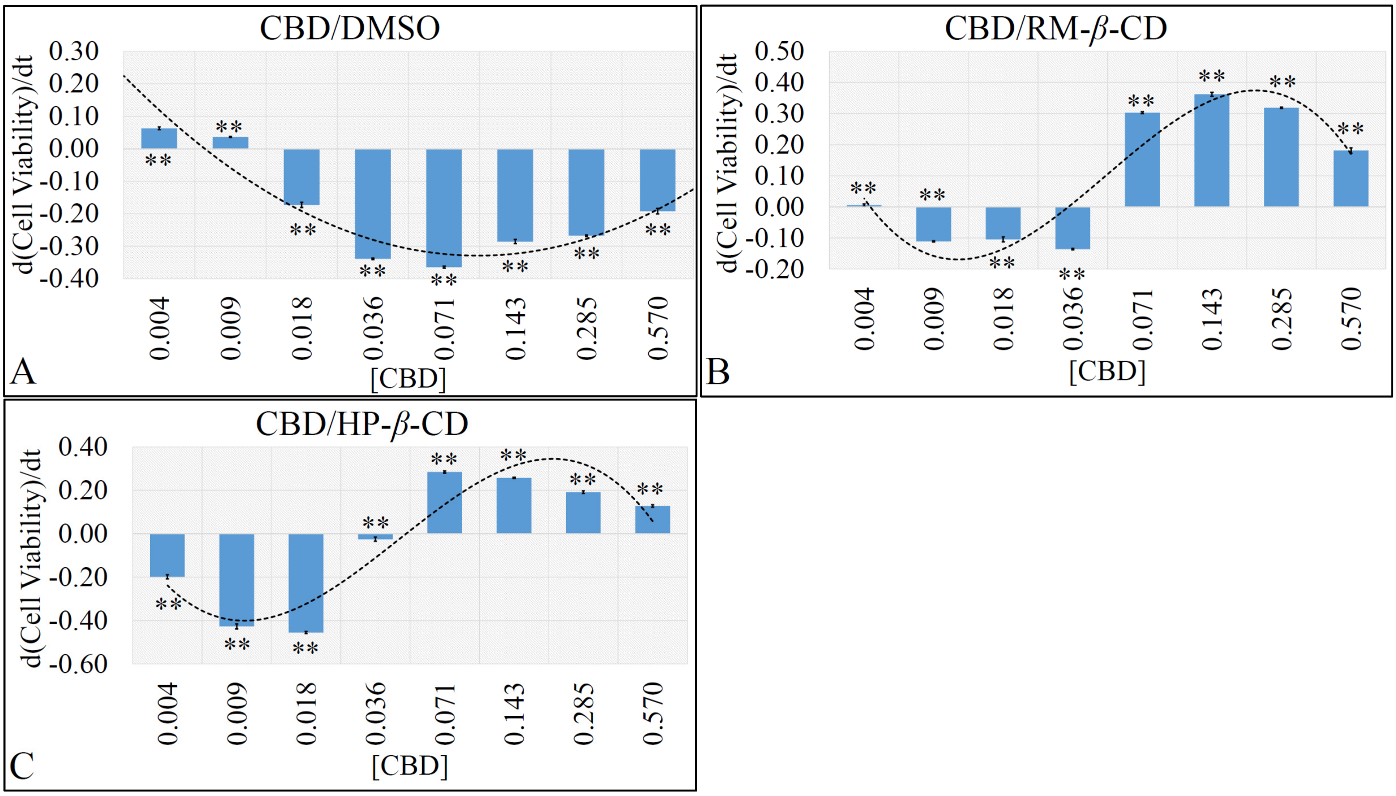


**Figure S10. The comparative recovery velocity of CBD complexes in the TE671 cells**. (**A**) Cells treated with CBD/DMSO did not recover all together and the observed differences were significant ($p < 0.001$). (**B**) Cells exposed to CBD/RM-*β*-CD manifested a cellular rescue at concentrations 0.071–0.57 mg/ml, whereas for the rest of the concentrations 0.004–0.036 mg/mL such rescue was not observed. (**C**) Cells treated with CBD/HP-*β*-CD manifested a similar behavior as the CBD/RM-*β*-CD complex, with a threshold at 0.036 mg/mL, in which they begun to recover. The asterisks ** depict a significance at the $p < 0.01$ level. The asterisks above all bars indicate that the differences are significant among all concentrations and in all combinations.

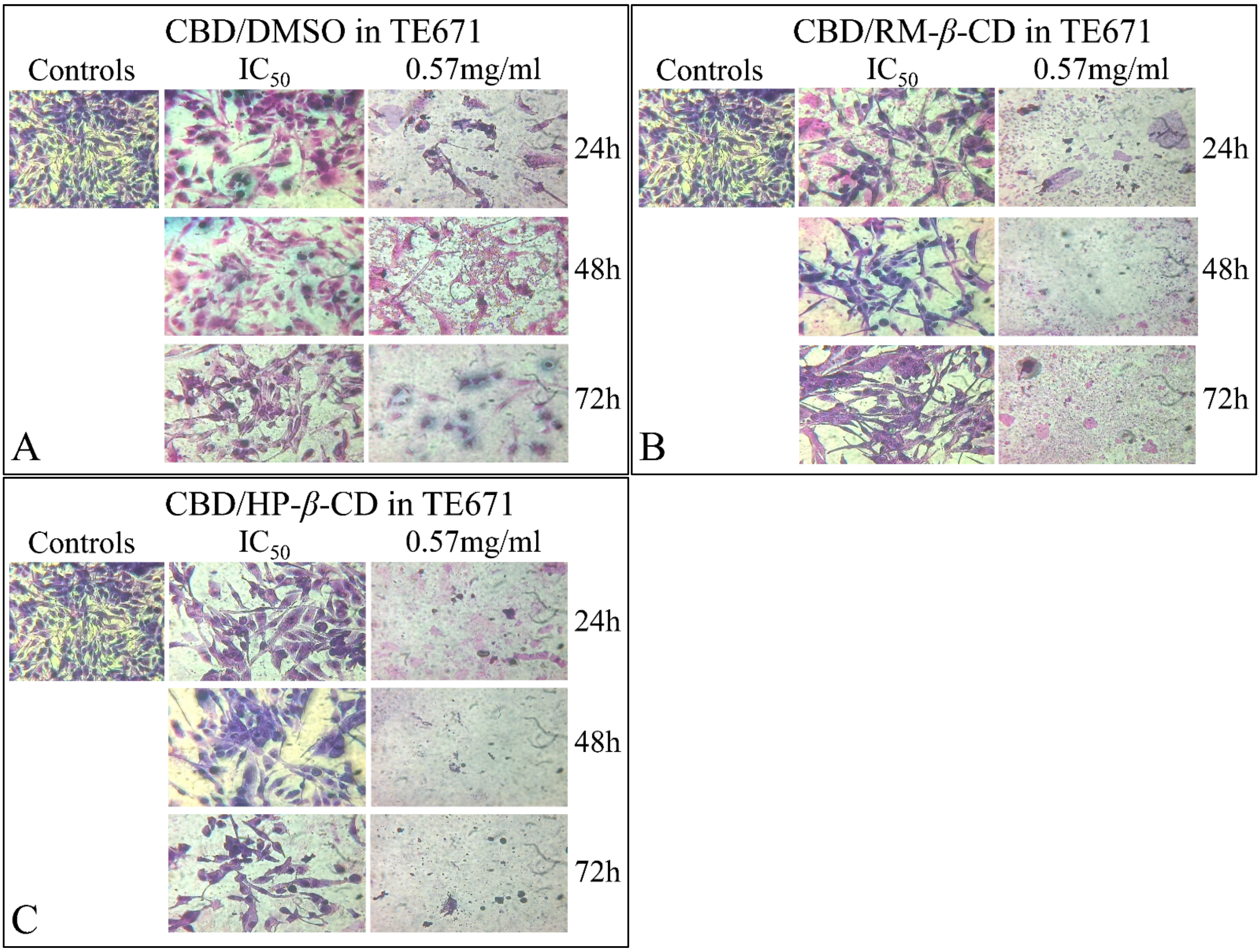


**Figure S11. Microscopic examination of TE671 cells exposed to CBD, and its inclusion complexes.** Microscopy confirmed the cell viability results for cells exposed to **(A)** CBD/DMSO, (**B**) CBD/RM-$\beta$-CD and **(C)** CBD/HP-$\beta$-CD inclusion complexes.
